# Health and economic impact of universal screening and management of alcohol use disorders in India: An economic modelling study

**DOI:** 10.1111/add.70469

**Published:** 2026-05-13

**Authors:** Neha Purohit, Shubhmohan Singh, Atul Ambekar, Ajay Duseja, Ashwani Kumar Mishra, Girish N. Rao, Kathirvel Soundappan, Atul Kotwal, Shankar Prinja

**Affiliations:** ^1^ Department of Community Medicine and School of Public Health Postgraduate Institute of Medical Education and Research (PGIMER) Chandigarh India; ^2^ Department of Psychiatry Postgraduate Institute of Medical Education and Research (PGIMER) Chandigarh India; ^3^ National Drug Dependence Treatment Centre All India Institute of Medical Sciences New Delhi India; ^4^ Department of Hepatology Postgraduate Institute of Medical Education and Research (PGIMER) Chandigarh India; ^5^ Centre for Public Health National Institute of Mental Health and Neurosciences Bangalore India; ^6^ National Health Systems Resource Centre New Delhi India

**Keywords:** alcohol‐related liver disease, alcohol use disorders, cost‐effectiveness, economic evaluation, head and neck cancers, primary health care, road traffic accidents, screening

## Abstract

**Aim:**

The study assessed the health and economic implications as well as the cost‐utility of implementing universal alcohol use disorder (AUD) screening in 15–74 years population at the primary healthcare level compared with the current practice of diagnosis and management of symptomatic AUD patients seeking formal healthcare.

**Design:**

Model‐based cost‐utility analysis using a hybrid model comprising a decision tree and lifetime age‐ and gender‐specific Markov models for alcohol attributable conditions, including road traffic accident injuries, alcohol‐related liver disease and head and neck cancers. The analysis was undertaken from both an abridged societal (consideration of direct cost of care) and a societal (consideration of direct and indirect costs) perspective.

**Setting:**

India (national and sub‐national level analysis).

**Participants:**

15–74 years population segregated by gender.

**Interventions and comparators:**

The intervention was 10‐year annual population‐based screening for alcohol use disorders using alcohol use disorder identification test by community health workers at primary care facilities. The comparator was ‘usual care’ scenario of diagnosis and management of symptomatic AUD patients, considering care seeking patterns in India.

**Measurements:**

Differences in life years, quality‐adjusted life years (QALYs), alcohol attributable deaths and morbidities, direct costs and indirect costs in the comparative scenarios, along with incremental cost‐utility ratio (ICUR), benefit–cost ratio and net monetary benefit. ICUR was evaluated using the per‐capita gross domestic product (GDP) threshold of ₹171 498 (US$2182), as per Indian economic evaluation guidelines. Probabilistic and deterministic sensitivity analysis was conducted to identify the parameters that are likely to have an impact on efficiency of the screening programme.

**Findings:**

The AUD universal screening programme was associated with a gain of 71.16 million QALYs at population level, with approximately one‐fourth reduction in the incidence of alcohol‐attributable conditions. The ICUR value indicated that the programme is likely to be cost‐effective from an abridged societal perspective. The intervention is projected to generate a gain of ₹8.21 (US$1.03) trillion, equivalent to per year gain of 0.59% of GDP, based on the abridged societal perspective. The deterministic sensitivity analysis indicated that reductions in diagnostic accuracy of the screening method, prevalence of AUD and treatment coverage had an inverse impact on the ICURs and could impact efficiency of the programme.

**Conclusion:**

There is good health and economic evidence to support the integration of alcohol use disorder screening and management within routine primary care. It would be essential to deploy measures for effectiveness of the screening tool and continuity of care to enhance efficiency of the programme.

## INTRODUCTION

Alcohol remains a significant psychoactive and dependence‐producing substance exerting a significant impact on global population health and development [1]. Despite the considerable public health consequences of alcohol use, it has often been relegated in policy discussions, with alcohol being the only psychoactive substance that lacks regulation under binding international legal instruments [2]. In high‐income countries (HICs), policies for regulating availability and providing preventive and therapeutic measures for alcohol use disorders (AUDs) have demonstrated effectiveness in mitigating harm [3, 4, 5]. However, the situation in low‐ and middle‐income countries (LMICs) is more complex, with a 2.8% annual growth rate of alcohol volume consumption per person, compared with 1.1% in HICs, and with riskier patterns of consumption [6, 7]. Further, protective factors, such as access to medical care or sin taxes on such products are often fewer, with constrained implementation of effective regulatory policies in LMICs.

At the health systems level, the World Health Organization’s Mental Health Gap Action Programme (mhGAP) Intervention Guide outlines three levels of intervention for managing AUDs: screening for the early detection of risky alcohol consumption, brief interventions by trained primary healthcare providers and specialist support for more complex cases [8]. Although screening and brief interventions have been shown to produce significant health benefits, they are among the most resource‐intensive interventions to implement [1, 7]. Moreover, the existing literature lacks economic evidence regarding the screening and management of AUDs in low‐income countries (LICs) and LMICs (Table S1) [1, 4, 5, 6].

India represents an illustrative case for examining the economic dimensions of health policies for reduction in alcohol consumption patterns and AUDs. Despite a relatively moderate national prevalence of alcohol use (15%), the country faces a disproportionately high burden of alcohol‐attributable morbidity and mortality owing to risky consumption patterns, with alcohol‐related deaths increasing markedly between 2000 and 2019 [9, 10]. India’s experience encapsulates several features typical of many countries across the world, in terms of socio‐economic transitions, increasing per capita alcohol consumption, substantial unrecorded production and consumption, and limited treatment access [11]. Moreover, ongoing healthcare reforms have created an opportunity to explore the integration of proactive AUD screening and management services at the primary care level, in line with the Indian Mental Health and Neurological Services (MNS) guidelines [12]. However, the implementation of these guidelines is currently in the nascent stage [13, 14]. At the same time, the country’s federal governance structure and diverse state‐level alcohol policies, ranging from complete prohibition to state monopolies and liberal licensing systems, creates a natural laboratory to study the impact of health policies on alcohol use and related health outcomes.

Therefore, using India as a case study for countries facing similar challenges, this study assesses the health outcomes and economic implications of implementing universal population‐level screening and management of AUDs at the primary point of contact, in Ayushman Arogya Mandir subcentres (SC‐AAMs) within the healthcare system, as compared with the current practice of management of symptomatic patients presenting for care.

## METHODOLOGY

### Overview of analysis

A mathematical model‐based analysis was conducted to assess the health and economic impact of annual population‐based screening using the Alcohol Use Disorder Identification Test (AUDIT) in a population of 15–74 year olds at SC‐AAMs, compared with the ‘usual care’ scenario, which reflects the current situation without a structured screening programme at primary healthcare (PHC) units in India (Figure S1). A lifetime analytical horizon was considered for assessing the long‐term impact of a 10‐year AUD screening and management programme, in view of the chronic nature of alcohol‐attributable conditions. The 10‐year implementation period was considered to evaluate the effectiveness of AUD treatment with or without proactive screening (measured in terms of individuals achieving abstinence) and associated screening and treatment costs [15, 16].

Consistent with the available Indian evidence, treatment efficacy was modelled as a transition to complete abstinence from alcohol consumption [17, 18, 19]. The selection of abstinence as the primary outcome was informed by contextual considerations and expert consultation with psychiatrists. While studies from HICs commonly report treatment effectiveness in terms of reductions in alcohol consumption [20, 21], such estimates may not be directly generalisable to LMIC settings, including India, owing to differences in baseline drinking patterns, health system capacity, treatment adherence and service delivery contexts [22]. Furthermore, clinical guidelines in India largely emphasise abstinence‐oriented management goals, supporting the relevance of this outcome measure [23, 24].

To capture the long‐term consequences of the intervention, health outcomes were measured over a lifetime analytical horizon in terms of quality‐adjusted life years (QALYs). Lifetime impact of the intervention was modelled through change in incidence of three major alcohol‐related health conditions: alcohol‐related liver disease (ALD), head and neck cancers (HNC) and injuries associated with road traffic accidents (RTA), which were selected for the particularly strong causal evidence linking alcohol consumption with the risk of their development [7, 25].

The analysis incorporated both health system costs and out‐of‐pocket expenses incurred under both usual care and screening scenarios, adopting an abridged societal perspective in line with the Indian methodological guidelines for economic evaluations [26]. As a scenario analysis, a societal perspective was used to include indirect costs, such as premature mortality and productivity losses owing to work hours lost, alongside the direct costs of formal healthcare.

In line with Indian economic evaluation standards, future costs and outcomes were discounted at an annual rate of 3%, and the strategy was considered cost‐effective if its incremental cost–utility ratio (ICUR), expressed as the incremental cost per QALY gained, was lower than the gross domestic product (GDP) per capita threshold of ₹171,498 ($2182) (₹ = Indian National Rupee) for the year 2021–22 [27].

Considering the wide variations in rates and patterns of alcohol consumption across Indian states [9], we also conducted a subnational analysis to determine the impact of universal screening on the relative reduction in number of cases and deaths associated with alcohol‐attributable conditions (ALD, HNC and RTA). As the economic analysis plan was not pre‐registered, the findings should be interpreted as exploratory.

### Model overview

**FIGURE 1:**
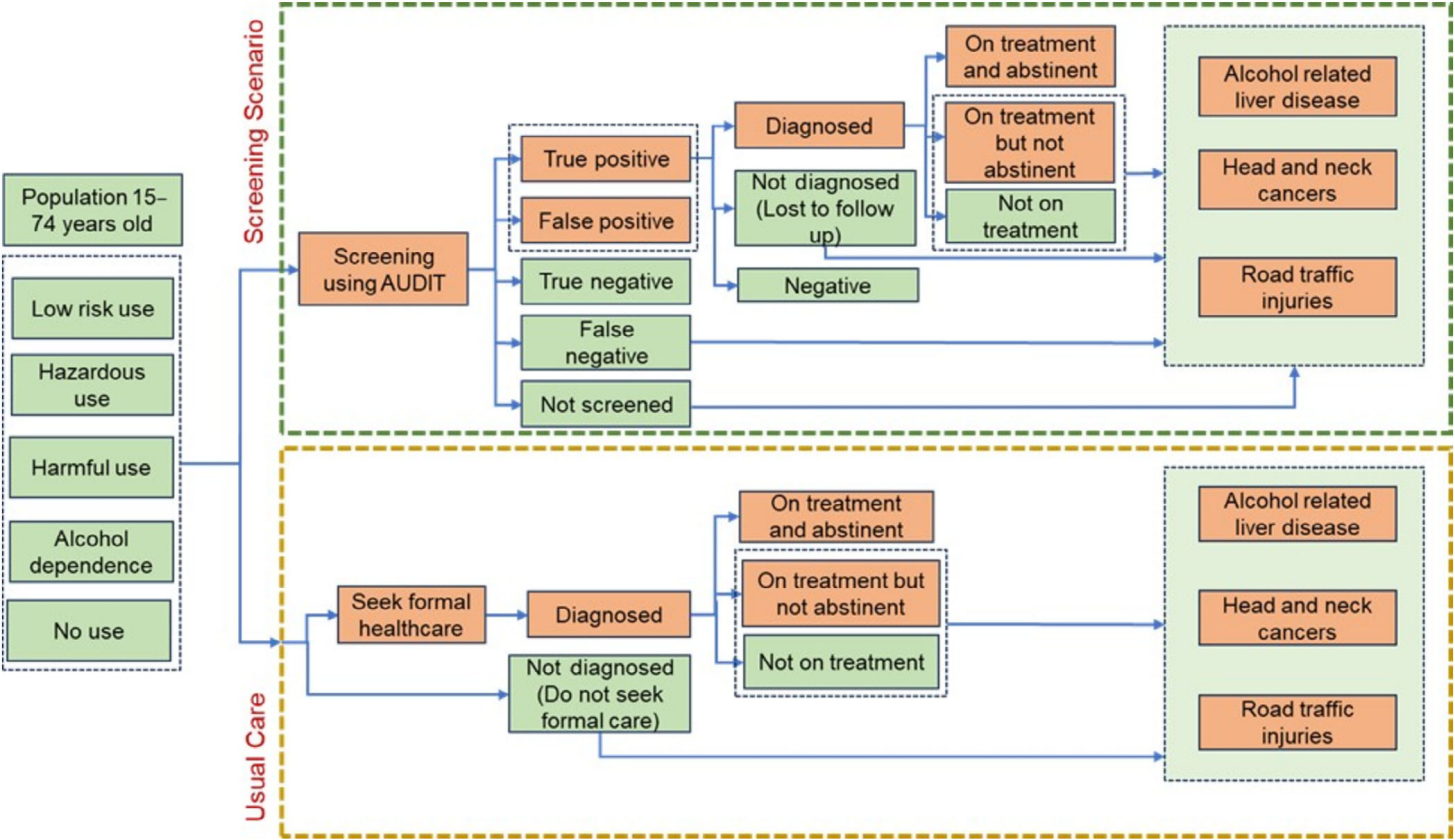
Schematic decision tree model. Alcohol use disorder (AUD) management: brief interventions for hazardous and harmful alcohol use. Psychosocial and pharmacological management for alcohol dependence. Orange boxes indicate cost‐drivers in the intervention and usual care. All the individuals who do not develop alcohol‐attributable conditions (alcohol‐related liver disease, head and neck cancers or road traffic accident injuries) in previous cycles will be eligible for annual screening for AUD in the subsequent years.

A two‐part mathematical model was developed in Microsoft Excel (Microsoft, Redmond, WA, USA). The first component consisted of a decision tree designed to estimate the reduction in incidence of alcohol use in each annual cycle (Figure 1). The second part involved the development of Markov state transition models for ALD, HNC, RTA and the population cohort with alcohol use but no comorbidities, to assess the health benefits and the associated costs attributable to the strategies in the two comparative scenarios over a lifetime horizon.

#### Part I: decision tree analysis

A decision tree analysis was conducted to assess the impact of an organised screening programme/usual care exclusively on the abstinence rate among consumers of alcohol, at the end of each year. The tree started with a baseline male and female population, based on the Indian census data for individuals aged 15–74 years in 2021 [28]. The population cohort was divided into eight categories based on sex assigned at birth and extent of alcohol use, using survey reports and Indian studies [9, 29, 30]. The age‐ and gender‐specific prevalence of alcohol consumption was calculated from the unit level data of the national survey on the Magnitude of Substance Use in India [9], which interviewed 473 569 individuals across 36 states and union territories using the WHO’s Alcohol, Smoking and Substance Involvement Screening Test **(**ASSIST) tool. The nationwide survey was also used to calculate the age‐ and gender‐ specific prevalence of AUD (people with harmful or dependent alcohol use). The proportions of the population with low‐risk and hazardous alcohol consumption were estimated by applying the corresponding proportions reported in cross‐sectional studies from India to the overall prevalence of alcohol consumption [29, 30] (Table S4). For subsequent years, we assumed that a new population of 15‐year‐olds will enter the model annually. Population growth was not factored into the model, given the short time horizon of the decision tree analysis.

The screening accuracy of the AUDIT tool was obtained from an Indian study using a cut‐off score of 8 [31]. The treatment rate in usual care scenario was based on findings from the National Mental Health Survey (NMHS) of India [32]. We assumed a 70% screening rate for the base‐case analysis. Additionally, a 15% loss to follow‐up was factored in for both the diagnosis of AUDs and subsequent treatment initiation (Table S2). It was also assumed that 66% of individuals who initiated treatment would become non‐compliant after 3 months [33].

The care‐seeking patterns for diagnosis and treatment in usual care were derived from the authors’ analysis of NMHS unit‐level data [32]. Patients were allocated to intervention pathways based on clinical severity using AUDIT score thresholds. Based on standard treatment guidelines in India, individuals with hazardous or harmful alcohol use were considered as managed through brief interventions alone, while those with alcohol dependence received treatments including psychosocial and pharmacological therapies (Table S3) [34, 35, 36]. The efficacy of brief intervention was obtained from a systematic review [37], while the efficacy of pharmacological treatments was obtained from randomised trials conducted in India (Table S3).

A relapse rate of 70% after 1 year was assumed after successful cessation, both in the absence and presence of a systematic screening programme [38, 39]. The annual number of individuals with alcohol use, by extent of use, who would be treated or remained untreated (those who do not seek treatment or remain non‐abstinent after formal care) in the 10‐year time period were calculated (Figure 1). Subsequently, the annual risks of developing ALD, HNC or RTA were applied to individuals in different categories of alcohol consumption at the end of each cycle.

#### Part II: Markov state transition models

To evaluate the lifetime costs and health outcomes associated with the development of ALD, HNC or RTA, and without the development of any of the three conditions, we developed 37 gender‐specific state transition models (Figures S3–S5). These models included 12 5‐yearly age‐specific models (from 15 to 75 years) each for HNC, RTA and a population cohort without any of the comorbid conditions. In contrast, a single Markov model was developed to simulate the natural progression of ALD, starting at a mean age of 40 years, owing to the lack of age‐specific incidence rates for ALD in India [40].

The input parameters—including state transition probabilities, age‐ and gender‐specific epidemiological parameters, treatment‐seeking parameters, utility parameters, and cost parameters—were derived from published literature and analyses of data from nationally representative surveys (Tables S4–S6). The age‐ and gender‐specific mortality rates were derived from the most recent Sample Registration Survey report [41]. Detailed descriptions of the individual Markov models are provided in the supporting information.

### Valuation of health consequences

We estimated the potential reduction in the incident cases and mortality associated with ALD, HNC and RTA in a population with alcohol use, after implementation of an organised screening programme for the 10‐year time frame. The overall LYs and QALYs were a sum total of the calculated LYs and QALYs for individuals with ALD, HNC, RTA and the population without disease conditions.

### Valuation of costs

The costs for ‘usual care’ were inclusive of the direct costs of the diagnosis and treatment of AUD for each year, as well as the lifetime costs pertaining to the diagnosis and treatment of ALD, HNC and RTA. Treatment costs included the cost of consultations, diagnostic tests, medicine/surgical costs and non‐medical direct costs (Tables S3–S6).

The screening scenario had an additional direct cost of screening at the SC‐AAMs (Figure S2; Table S2), which was calculated using parameters sourced from the National Health System Cost Database of India (NHSCD), which compiles service delivery cost information from over 200 primary healthcare (PHC) facilities and more than 80 public and private health facilities [42]. Diagnostic and treatment costs were sourced from the national health insurance scheme (Central Health Government Scheme rates and Pradhan Mantri Jan Arogya Yojana package rates) and the NHSCD [42, 43]. The costs were calculated in Indian national rupees (₹) and adjusted for inflation to reflect the valuation in 2022. All costs were converted to US dollars, using 2022 conversion rate (1 $ = ₹78.60) [44].

The indirect costs were calculated using the GDP per capita based human capital approach [45]. The indirect costs were a sum of the costs of premature mortality and the loss of productivity attributable to diseases in patients with alcohol use.

### Economic analysis of the two scenarios

We computed the ICUR by dividing the difference in discounted costs by the difference in discounted QALYs among the alternative comparative scenarios. Subsequently, we derived the net monetary benefits (NMB) by the subtraction of incremental costs from net monetised QALYs. One QALY was equivalent to per capita GDP of the country.

Probabilistic sensitivity analysis was conducted to address the joint uncertainty in the input parameters. The key parameters were assigned appropriate probability distributions based on their characteristics (Tables S3–S6). We performed 10 000 Monte Carlo simulations to determine the probability of the intervention being cost‐effective. Additionally, a deterministic sensitivity analysis was conducted to evaluate the impact of variations in individual parameters on the ICUR values.

We also assessed the cost‐effectiveness of the screening programme using prevalence estimates of alcohol use from the National Family Health Survey 5 (NFHS‐5) instead of the ‘national level survey on Magnitude of Substance Use in India’, used in the base‐case analysis [9, 46]. A scenario analysis was conducted to evaluate the economic impact of the screening strategy when limited to individuals aged 30–74 years, aligning with the target age group for screening other mental health disorders.

### Ethical approval

The research study was approved by the Institutional Ethics Committee, Post Graduate Institute of Medical Education and Research, Chandigarh, India (PGI/IEC/2024/1599).

## RESULTS

### Impact on health outcomes

Implementing population‐level screening in India is projected to result in a gain of 57.78 million LYs and 71.16 million QALYs (Tables 1 and S7). This equates to a gain of 0.35 LYs and 0.43 QALYs per individual with alcohol use. Compared with current practice, the implementation of screening and management is anticipated to lead to a relative reduction in the incidence of alcohol‐attributable RTA, HNC and ALD by 31%, 30% and 23%, respectively (Table 2). At the population level, this corresponds to an absolute reduction of 2.77 percentage points in the burden of alcohol‐attributable conditions (Table S8). In addition, the intervention is projected to avert 2.3 million alcohol‐related deaths attributed to ALD, HNC and RTA, representing a 24.2% relative reduction in alcohol‐related mortalities compared with the status quo.

**TABLE 1:** Discounted life years and quality‐adjusted life years (QALYs) in usual care and in the screening scenario.

	Life years (billions) (95% CI)	QALYs (billions) (95% CI)	Life year gain per individual with alcohol use (95% CI)	QALY gain per individual with alcohol use (95% CI)	Life year gain per capita (95% CI)	QALY gain per capita (95% CI)
Usual care	4.359 (4.354–4.364)	4.298 (4.292–4.304)				
Screening programme	4.417 (4.412–4.422)	4.369 (4.363–4.375)	0.347 (0.340–0.353)	0.427 (0.419–0.435)	0.048 (0.047–0.048)	0.059 (0.058–0.060)

**TABLE 2:** Number and percentage relative reduction in incident cases and deaths.

Alcohol‐attributable disease conditions	Number of cases^a^		% reduction	Number of mortalities^a^		% reduction
	Usual care	Screening		Usual care	Screening	
Road traffic accident injuries	3.13	2.15	31%	1.59	1.09	31%
Head and neck cancers	0.38	0.27	30%	0.04	0.02	41%
Alcohol‐related liver disease	142.76	110.46	23%	7.89	6.10	23%
Total	146.27	112.87	23%	9.51	7.21	24%

^a^ Numbers are rounded off according to the value at the second decimal place, in millions.

**FIGURE 2:**
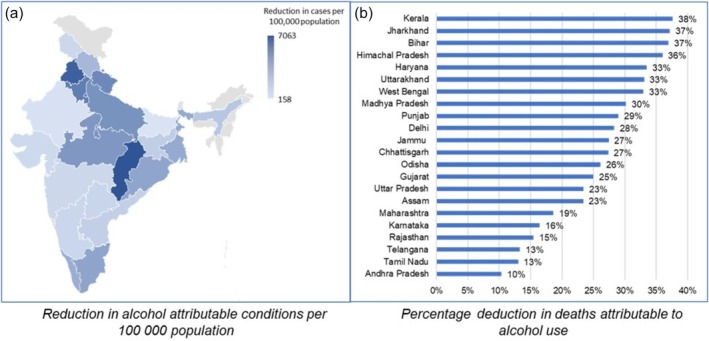
Impact of alcohol use disorder (AUD) screening at the subnational level in terms of reduction in alcohol‐attributable conditions (a) and associated mortalities (b).

We found that the universal screening for AUD will have higher reductions in incident cases of alcohol‐attributable conditions per 100 000 population in Chhattisgarh, and the northern states of Punjab, Haryana and Delhi (Figure 2; Table S9).

### Direct and indirect costs

The 10‐year screening programme would incur an additional discounted cost of ₹3.4 ($0.04) trillion, equating to a per capita annual cost of ₹282 ($3.5) (Table 3). Tables S10 and S11 present the undiscounted and discounted direct costs for the two scenarios. In terms of indirect costs, discounted savings of ₹6914 ($88) billion are projected, respectively, resulting from a reduction in premature mortality and the loss of productive days (Tables 4, S12 and S13). When considering both direct and indirect costs, the screening programme is expected to lead to a saving of ₹3510 ($44) billion (0.2% GDP per year) at the population level, which corresponds to a per capita annual saving of ₹ 291 ($4).

**TABLE 3:** Discounted direct costs with and without alcohol use disorder (AUD) screening (in ₹ billions).

Cost components	Usual care^a^	Screening programme	Difference between usual care and screening programme
Screening and treatment of AUD	1472 (1458–1487)	8670 (8592–8749)	7198 (7134–7262)
Treatment of road traffic accident injuries	207 (204–210)	141 (139–142)	−66 (65–66)
Treatment of head and neck cancers	209 (208–211)	136 (135–137)	−73 (72.5–73.5)
Treatment of alcohol‐related liver disease	16 954 (16 793–17 115)	13 299 (13 165–13 433)	−3655 (3627–3682)
Total direct costs	18 842 (18 681–19 004)	22 247 (22 097–22 397)	3404 (3328–3480)

^a^ No screening costs. Table S11 presents the values in US$.

**TABLE 4:** Indirect costs (billions ₹) associated with premature mortality and loss of productivity.

	Indirect costs			Total
	RTA	HNC	ALD	
Usual care	5362 (5332‐5392)	2629 (2615‐2643)	20 089 (20 000‐20 177)	28 080 (27 985‐28 176)
Screening	3666 (3643‐3688)	1700 (1689‐1710)	15 801 (15 718‐15 884)	21 166 (21 071‐21 261)
Indirect Costs averted	1697 (1688‐1704)	929 (925–934)	4288 (4283‐4293)	6914 (6854–6974)

Abbreviations: ALD = alcohol‐related liver disease; HNC = head and neck cancers; RTA = road traffic accident injuries. Table S13 presents the values in US$.

### Economic evaluation results

From an abridged societal perspective, the screening leads to an incremental cost of ₹47,890 ($609) per QALY gained, which is below the GDP per capita threshold. From a societal perspective, the intervention was found to be cost‐saving. In terms of NMB, the intervention is projected to generate a gain of ₹8.80 trillion in a 10‐year period (per year gain of 0.59% of GDP), based on the abridged societal perspective. When accounting for the additional indirect costs saved through reduced premature mortality and productivity losses associated with alcohol‐attributable diseases, the total gain is estimated to be ₹15.71 ($0.2) trillion (per year gain of 1.05% of GDP). The probability of screening being cost‐effective below the GDP per capita threshold was 99% and 100%, from abridged societal and societal perspectives, respectively.

The use of prevalence data from the NFHS‐5 resulted in lower LY and QALY gain, in comparison with the base‐case analysis (Table S14). However, the screening was found to be cost‐effective (ICUR = ₹100,608, $1280) from an abridged societal perspective, and cost‐saving (ICUR = –₹21,900, −$278) from a societal perspective. Similarly, limiting the screening intervention to the population aged over 30 years also proved to be cost‐effective (Table S15). However, this approach resulted in lower health benefits (0.03 billion QALY gains) than the screening strategy for the 15–74 years age group (0.07 billion QALY gains).

A few parameters had a noteworthy impact on the findings of economic evaluation. Specifically, if the prevalence of alcohol use decreased by 58% from its current level, or if the sensitivity of the AUDIT tool is reduced from 93% to 45% (52% reduction in true positives), the universal screening strategy is no longer cost‐effective. Moreover, the treatment rate and patient compliance with treatment following screening and diagnosis also influenced the cost‐effectiveness results (Table S16). If fewer than 24% of diagnosed AUD patients received treatment, or if treatment compliance falls below 21%, the screening would cease to be an economically viable strategy. Conversely, if the relapse rate can be reduced to 21% without any additional cost implications, the intervention becomes cost‐saving.

### Model validation

The model‐generated estimates of LYs at various ages within the population with alcohol use, excluding other comorbidities, were found to closely align with the national estimates from the Sample Registration System (SRS) life tables (Table S17) [41]. Additionally, the model‐based median overall survival for HNC was analysed to be 6 years, consistent with existing epidemiological literature [47]. Similarly, the model findings on the median age of onset of hepatocellular carcinoma in patients with ALD were in accordance with the literature, with a median age of 59 years for males and 61 years for females, and with a median survival of 1 year [48, 49].

## DISCUSSION

Our analysis provides robust health and economic evidence supporting the adoption of a universal screening programme for AUD in the population aged 15–74 years, utilising the AUDIT at SC‐AAMs in India. As reported in other international studies, we also found that the early detection and management of AUD is likely to be cost‐effective, even under pessimistic assumptions [15, 16, 50, 51, 52]. Furthermore, the incremental benefit–cost ratio of 4.6 : 1 (from a societal perspective) closely aligns with the findings from studies on AUD screening and management in PHC settings (4.3 : 1) [53], as well as findings from the emergency department (3.81 : 1), indicating a high return on the investment [54]. The findings also align with previous literature indicating the cost‐effectiveness of screening programmes for other non‐communicable diseases at the primary care level in India [55, 56].

The integration of universal AUD screening within PHC settings is projected to yield gains of 57.78 million LYs and 71.16 million QALYs at the population level. In comparison with the findings of Jyani *et al*., who estimate a gain of 258 million LYs and 552 million QALYs assuming a reduction in alcohol consumption to zero in India [25], our real‐world analysis suggests that screening and management can contribute to 22% and 13% of the LY and QALY gains, respectively. The subnational analysis suggests that when adjusted for population size, the states of Chhattisgarh, Punjab, Haryana and Delhi are likely to benefit more from a universal screening programme in terms of a reduction in alcohol‐attributable morbidities and mortalities.

There is a significant heterogeneity in model‐based estimated gains in QALYs reported in different studies [15, 16, 50]. Much of these differences emanate from differences in methodology. For instance, Zur and Zariac used similar age‐segregated QALY weights across a range of health conditions, based on differences in alcohol consumption pattern in the Canadian community, while Solberg *et al*. used the same QALY weights for acute and chronic conditions, based on assumptions [50, 51]. While these may be the best guesses to initiate the process of the assessment of important public health outcome indicators, our study is more robust in terms of the methodological application of disease‐related and severity‐related quality‐of‐life weights. Our results of incremental LYs and QALYs resemble those reported by Zur and Zariac from Canada (Table S18) [50]. However, our estimates of QALYs gained were higher in comparison with studies conducted in Europe by Angus *et al*. and with other studies applying a shorter time horizon for the economic analysis [15, 16].

The shares of direct and indirect economic costs of alcohol consumption in the usual care scenario were 39% and 61%, respectively, similar to findings of the systematic review [57]. The gross macro‐economic impact of the intervention was significant, with a per year gain of 0.55% and 1.02% of GDP, based on the abridged societal and societal perspective, respectively. An earlier systematic review had concluded that the economic cost of negative (health) consequences of alcohol consumption is equivalent to 1.5%–2.6% GDP [57].

We found that the universal screening programme ceases to be cost‐effective if the sensitivity of the AUDIT tool falls below 45%. Most of the published studies have found the sensitivity of the tool to be more than 60% in primary care settings; however, Morton *et al*. found the sensitivity to be 33% in the older population [58, 59]. Furthermore, there is growing evidence on the effectiveness of non‐specialist health workers in delivering frontline interventions for AUD in LMICs [60]. However, the implementation barriers on the supply side are primarily related to the capacity‐building needs of healthcare providers and the low engagement of providers in discussions on alcohol consumption [61]. This underscores the need of effective training for the screening and management of AUD to ensure adequate pick‐up rates, thus assuring the efficiency of the programme.

Screening for AUD alone is not a cost‐effective strategy, unless it is effectively integrated with management, similar to findings in the context of other non‐communicable diseases in India [55]. Specifically, if fewer than 24% of patients diagnosed with AUD received treatment, universal screening would not be economically viable. Our analysis further suggests that even with a high treatment initiation rate (up to 85%), the screening strategy would be cost‐ineffective if treatment compliance falls below 21% at the population level. In light of current care‐seeking patterns, where only 13% of individuals with AUD access formal treatment services and where adherence to treatment remains low [32], these findings highlight the criticality of integrated care models and sustained efforts to ensure continuity of care.

We further highlight that changes in the prevalence of alcohol consumption (to less than 8%) would warrant a reassessment of the policy. This indicates that screening limited to females would be cost‐ineffective in the current scenario. However, the recent evidence indicates that alcohol consumption among women in India is rising steadily, narrowing the gender gap observed in earlier years [62]. Moreover, women who consume alcohol face a higher risk of developing alcohol‐attributable health conditions compared with men and experience greater stigma and barriers to care‐seeking within the Indian context [63]. Consequently, considering a screening scenario restricted to men, which would increase the efficiency of the programme, is neither pragmatically nor ethically justifiable, given the public health and equity implications.

### Strengths and limitations

The economic assessment of universal screening for AUD in an LMIC setting is novel and has notable strengths. Almost all the published cost–utility studies from HICs have computed the reduction in QALYs using expected life expectancy in the presence of disease conditions and the standard QALY weights for chronic and acute illnesses [15, 16, 50, 51]. Furthermore, the calculation of healthcare costs is less complicated in HICs with well‐established electronic health systems. However, in LICs and LMICs, where the evidence on QALYs lost through specific diseases and the information on lifetime cost of treatment are in a nascent stage, adopting such a methodology is not feasible. We, therefore resorted to a more nuanced methodology of developing three lifetime models to derive the lifetime QALYs and costs associated with alcohol‐attributable diseases. Such an approach may be followed by regions with similar data availability challenges, until standardized information systems are developed for capturing health information and cost of care.

We further derived the model parameters from nationally representative surveys on prevalence and patterns of alcohol consumption (Magnitude of Substance Use in India), healthcare‐seeking patterns (NMHS), costs associated with different input resources for consultations and management of diseases in public and private sector facilities (NHSCD) and out‐of‐pocket expenditures (National Sample Survey) [9, 32, 41, 42, 43, 64]. The consideration of demographic factors, such as age and gender at subnational and national levels adds robustness to the estimates. We used the CHEERS checklist to ensure transparency in the reporting of the economic evaluation (Table S19) [65].

We acknowledge certain limitations to the study. First, alcohol consumption is associated with several disease conditions. We chose to model the diseases that have well‐established and strong associations with alcohol use [66]. This makes our estimates conservative, and the cost‐effectiveness of the intervention is likely to increase with the inclusion of other diseases that can be prevented through screening and the appropriate management of AUD.

Second, drawing on evidence from India, we used the efficacy of AUD management as transitioning to complete abstinence from alcohol use. Non‐consideration of the impact of interventions on reductions in alcohol consumption means that our results are more conservative. Third, we did not account for the residual risk of alcohol‐attributable conditions that may persist following abstinence. However, as the risks of developing ALD (in the absence of prior hepatic damage) and RTAs become negligible once abstinence is achieved, and as these constitute the major contributors of the overall disease burden in the analysis, the exclusion of the residual risk for development of HNC among abstinent individuals is unlikely to affect the overall direction of the findings.

Fourth, the diagnostic accuracy of AUDIT was considered the same for both males and females, and consistent across age groups, in view of data limitations. While the evidence used for this model parameter did not impose any exclusion criteria related to age or gender, there could be differences in the accuracy of the tool based on demographic characteristics, and this calls for future epidemiological research. Fifth, we assumed similar treatment efficacy, irrespective of previous receipt of treatment, owing to data limitations.

Sixth, identical treatment uptake and adherence rates were applied to both genders in the analysis, reflecting the mixed‐gender composition of the underlying evidence base. In practice, however, women are likely to encounter greater barriers to accessing treatment, particularly under the usual care scenario; however, such disaggregated quantitative data remain scarce. Nonetheless, this limitation is unlikely to alter the study conclusions, as the applied probabilities represent mean estimates derived from mixed‐gender samples.

We did not account for any loss of productivity in ‘alcohol use without comorbidity’ or for caregivers of the patients with alcohol‐attributable conditions, owing to data unavailability. Therefore, we believe that one may anticipate higher gains in a real‐world scenario than the estimates from present analysis. Finally, the state‐level analysis incorporating different rates of alcohol use and AUD, assumes uniform healthcare utilisation in the usual‐care scenario across the states. While there could be differential access to healthcare across states, we consider that the observed prevalence of AUD partially reflects these disparities in access. Consequently, we expect the relative impact of interventions to follow similar patterns across states, as found in the analysis. However, this assumption merits further investigation.

In terms of generalisability, our model would prove an adaptable framework for other resource‐constrained economies with similar epidemiological settings to India. However, a range of adaptations would be necessary, dependent on differences in terms of drinking patterns and health‐seeking behaviours of the population.

## CONCLUSION

Given the projected health and economic benefits, the universal AUD screening programme at an early age of 15 years within the primary healthcare framework is a compelling investment case for policy adoption in LMICs such as India. It is crucial to invest in building the capacity of primary healthcare teams, integrating AUD screening and management within routine primary care, and ensuring continuity of care to foster long‐term compliance to ensure the efficiency of the intervention.

## AUTHOR CONTRIBUTIONS

**Neha Purohit:** Conceptualization (lead); data curation (lead); formal analysis (lead); methodology (lead); software (lead); supervision (equal); writing—original draft (lead); writing—review and editing (lead). **Shubhmohan Singh:** Conceptualization (supporting); data curation (supporting); formal analysis (supporting); methodology (equal); supervision (equal); validation (lead); visualization (equal); writing—review and editing (equal). **Atul Ambekar:** Data curation (equal); formal analysis (equal); methodology (equal); supervision (supporting); validation (lead); visualization (equal); writing—review and editing (equal). **Ajay Duseja:** Data curation (supporting); methodology (supporting); validation (equal); visualization (equal); writing—review and editing (equal). **Ashwani Kumar Mishra:** Data curation (supporting); formal analysis (equal); validation (equal); visualization (equal); writing—review and editing (equal). **Girish N. Rao:** Formal analysis (supporting); validation (equal); visualization (equal); writing—review and editing (equal). **Kathirvel Soundappan:** Conceptualization (supporting); methodology (supporting); supervision (supporting); validation (equal); visualization (equal); writing—review and editing (equal). **Atul Kotwal:** Conceptualization (supporting); methodology (supporting); supervision (supporting); validation (equal); visualization (equal); writing—review and editing (equal). **Shankar Prinja:** Conceptualization (lead); funding acquisition (lead); methodology (lead); project administration (lead); resources (lead); supervision (lead); validation (lead); visualization (lead); writing—review and editing (equal).

## DECLARATION OF INTERESTS

The authors have nothing to declare that is relevant to the content of this article.

## Supporting information


**Figure S1.** Comparative scenarios.
**Figure S2.** Details of cost components in screening scenario.
**Figure S3.** Markov state transition model for road traffic accident injuries.
**Figure S4.** Markov state transition model for alcohol‐related liver disease.
**Figure S5.** Markov state transition model for head and neck cancers.
**Table S1.** Summary of economic evaluations of management strategies for alcohol use disorders.
**Table S2.** Per person cost of AUDIT based screening according to variations in screening coverages (₹).
**Table S3.** Base value of parameters for decision tree analysis and data sources.
**Table S4.** Base value of parameters used for Markov model on road traffic accident injuries and data sources.
**Table S5.** Base value of parameters for Markov model on alcohol‐related liver disease and data sources.
**Table S6.** Base value of parameters for Markov model on head and neck cancers and data sources.
**Table S7.** Undiscounted life years and QALYs in usual care and screening scenario.
**Table S8.** Incident cases of alcohol‐attributable conditions at population level (%).
**Table S9.** State‐wise reduction in alcohol‐attributable conditions.
**Table S10.** Undiscounted direct costs (in ₹ billions).
**Table S11.** Discounted direct costs with and without AUD screening (in $ billions).
**Table S12.** Undiscounted indirect costs (in ₹ and $ billions).
**Table S13.** Indirect costs (in $ billions) associated with premature mortality and loss of productivity.
**Table S14.** Discounted life years, QALYs, costs and ICURs, using NFHS‐5 prevalence data.
**Table S15.** Discounted life years, QALYs, costs and ICURs, if screening is conducted in 30–74 years age group.
**Table S16.** Impact of varying rates of treatment and treatment compliance on ICUR values (₹)*.
**Table S17.** Comparison of life years of people with alcohol use without comorbidity from the model with SRS life tables.
**Table S18.** Comparison of discounted life year and QALY gain attributable to screening interventions for alcohol use disorders.
**Table S19.** Compliance with CHEERS checklist [148].

## Data Availability

The data used in the analysis has been provided in the manuscript and the supplementary material.
