# Supplementary material for: Health and economic impact of universal screening and management of alcohol use disorders in India: An economic modelling study
Source: Addiction. 2026 May 13;121(9):2341–52. doi: 10.1111/add.70469 (PMC13486922; doi:10.1111/add.70469)
Supplement: Supplementary file 1 [file ADD-121-2341-s001.docx]

**Supplementary material**

**Health and economic impact of universal screening and management of alcohol use disorders in India: An economic modelling study**

Neha Purohit, Shubhmohan Singh, Atul Ambekar, Ajay Duseja, Ashwani Kumar Mishra, Girish N Rao, Kathirvel Soundappan, Atul Kotwal, Shankar Prinja

**Table of Contents**

[**Section 1.** Summary of economic evaluations of management strategies for Alcohol Use Disorders	3](#_Toc225963424)

[**Section 2. Background and Description of screening strategy**	18](#_Toc225963425)

[**Section 3**. Methods for costing of screening services for alcohol use disorders	19](#_Toc225963426)

[Break-up of costs for implementation of alcohol use disorder screening programme	21](#_Toc225963427)

[**Section 4**. Model related structures and parameters	22](#_Toc225963428)

[**Section 4A. Decision Tree Analysis**	22](#_Toc225963429)

[**Section 4B. Markov state transition models**	31](#_Toc225963430)

[*Road traffic Accident Injuries: Model structure and parameters*	31](#_Toc225963431)

[*Alcohol related liver disease (ALD): Model structure and parameters*	37](#_Toc225963432)

[Head and Neck Cancers: Model structure and parameters	41](#_Toc225963433)

[**Section 5. Results**	45](#_Toc225963434)

[**Section 6. References**	52](#_Toc225963435)

**List of Figures**

[Figure S1. Comparative scenarios	19](#_Toc225963436)

[Figure S2. Details of cost components in screening scenario	21](#_Toc225963437)

[Figure S3. Markov state transition model for Road Traffic Accident Injuries	31](#_Toc225963438)

[Figure S4. Markov state transition model for Alcohol Related Liver Disease	37](#_Toc225963439)

[Figure S5. Markov state transition model for Head and Neck Cancer	42](#_Toc225963440)

**List of Tables**

[Table S1. Summary of economic evaluations of management strategies for Alcohol Use Disorders	3](#_Toc225963405)

[Table S2. Per person cost of AUDIT based screening according to variations in screening coverages (₹)	21](#_Toc225963406)

[Table S3. Base value of parameters for decision tree analysis and data sources	22](#_Toc225963407)

[Table S4. Base value of parameters used for markov model on Road traffic accident injuries and data sources	32](#_Toc225963408)

[Table S5. Base value of parameters for markov model on Alcohol Related Liver Disease and data sources	38](#_Toc225963409)

[Table S6. Base value of parameters for markov model on Head and Neck Cancer and data sources	42](#_Toc225963410)

[Table S7. Undiscounted life years, and QALYs in usual care and screening scenario	45](#_Toc225963411)

[Table S8. Incident cases of alcohol attributable conditions at population level (%)	46](#_Toc225963412)

[Table S9. State-wise reduction in alcohol attributable conditions	46](#_Toc225963413)

[Table S10. Undiscounted Direct costs (in ₹ billions)	46](#_Toc225963414)

[Table S11. Discounted direct costs with and without AUD screening (in $ billions)	47](#_Toc225963415)

[Table S12. Undiscounted Indirect costs (in ₹ and $ billions)	47](#_Toc225963416)

[Table S13. Indirect costs (billions $) associated with premature mortality and loss of productivity	48](#_Toc225963417)

[Table S14. Discounted life years, QALYs, costs and ICURs, using NFHS-V prevalence data	48](#_Toc225963418)

[Table S15. Discounted life years, QALYs, costs and ICURs, if screening is conducted in 30-74 years age group	48](#_Toc225963419)

[Table S16. Impact of varying rates of treatment and treatment compliance on ICUR values (₹)*	49](#_Toc225963420)

[Table S17. Comparison of life years of people with alcohol use without co-morbidity from the model with SRS life tables	49](#_Toc225963421)

[Table S18. Comparison of discounted life year and QALY gain attributable to screening interventions for alcohol use disorders	50](#_Toc225963422)

[Table S19. Compliance to CHEERS Checklist [148]	50](#_Toc225963423)

# **Section 1.** Summary of economic evaluations of management strategies for Alcohol Use Disorders

Table S1. Summary of economic evaluations of management strategies for Alcohol Use Disorders

| S. No | Author | Country/ Perspective | Intervention | Comparator | Study design | Time horizon (months)/ Discount rate | Measure of benefits | Conclusion |
| --- | --- | --- | --- | --- | --- | --- | --- | --- |
| 1. | Barbosa et al (2017) [1] | USA/ Healthcare | Brief Intervention within SBIRT Programme | Brief Treatment within SBIRT Programme | CEA | NR/ NR | Alcohol consumption, Treatment uptake |  |
| 2. | Barrett et al (2016) [2] | United Kingdom/ Societal | Brief Intervention | Information Only | RCT, CEA | 12/ 2% | Alcohol consumption |  |
| 3. | Hunter et al (2017) [3] | Italy/ Healthcare | Face-To-Face Brief Intervention | Facilitated Access to Website | RCT, CUA | 12/  NR | QALY |  |
| 4. | Kouimtsidis et al (2017) [4] | United Kingdom/ Healthcare | Extended Brief Interventions | Usual Care | RCT, CUA | 3/ NR | Alcohol consumption, QALY |  |
| 5. | Li et al (2017) [5] | USA/ Payer | Brief Alcohol Intervention (Alcohol Misconduct Prevention Program) | Do Nothing | CEA, CBA | 27/NR | Alcohol related incidents, monetary valuation of incidents avoided |  |
| 6. | Mundt et al (2005) [6] | USA/ Societal | Brief Intervention | General Health Booklet | RCT, CBA | 24/ 5% | Costs associated with hospital stays, emergency department visits |  |
| 7. | Shakeshaft et al (2020) [7] | Australia/ Healthcare | Brief Intervention | CBT | RCT, CEA | 6/ NR | Alcohol consumption |  |
| 8. | Watson et al (2015) [8] | United Kingdom/ Societal | Brief Intervention (By Occupational Health Nurse) | No Brief Intervention | RCT, CUA | 6/ NR | Alcohol consumption, QALY |  |
| 9. | Wutzke et al (2001) [9] | Australia/ Healthcare | Control Strategy (No Training and support) | Do Nothing | CEA | Lifetime/  3% | Life years |  |
|  |  |  | No-Support Strategy (training for five minutes, with no provision for follow-up assistance) | Control Strategy |  |  |  |  |
|  |  |  | ‘Minimal-Support’ Strategy (introductory training (five minutes) followed by fortnightly guidance sessions for receptionists) | No Support Strategy |  |  |  |  |
|  |  |  | ‘Maximal-Support’ Strategy (introductory training (five minutes) followed by a schedule of alternating phone and in-person check-ins every two weeks) | No Support Strategy |  |  |  |  |
| 10. | Humphreys et al (1996) [10] | USA/ Healthcare | Alcoholics Anonymous | Professional Outpatient Treatment | RCT, CEA | 36/ 5% | Treatment uptake |  |
| 11. | Agus et al (2019) [11] | United Kingdom/ Societal | The Steps Towards Alcohol Misuse Prevention Program (Stampp) | Normal Education  (no intervention) | RCT, CEA | 33/ 3.50% | Heavy episodic drinking |  |
| 12. | Drost et al (2016) [12] | Netherlands/ Healthcare | Web-delivered interactive game providing customized alcohol-related feedback for teenagers | Usual care | RCT, CEA | NR/  NR | Alcohol Consumption |  |
| 13. | Fleming et al (2020) [13] | USA/ Societal | Brief Physician Advice | Control Group | RCT, CEA, CBA | 4/  NR | Alcohol consumption, Alcohol related events, Treatment costs |  |
| 14. | Lindholm et al (1998) [14] | Sweden/ Societal | Five general practitioner visits for 1 year | 25 general practitioner visits for 5 years | RCT, CBA | 48/  NR | Life Years |  |
| 15. | Nadkarni et al (2017) [15] | India/ Healthcare | Counselling plus enhanced usual care | Enhanced usual care alone | RCT, CUA | 12/ 5% | Remission, QALY |  |
| 16. | Goetzel et al (2014) [16] | USA/ Societal | Comprehensive Worksite Health Promotion Programme | Doing Nothing | RCT, CBA | 12/  NR | Medical and productivity costs |  |
| 17. | Sobell et al (2002) [17] | USA/ Healthcare | Bibliotherapy/ Drinking Guidelines | Motivational Enhancement | RCT, CEA | 12/  NR | Alcohol consumption, Alcohol related events |  |
| 18. | Månsdotter et al (2007) [18] | Sweden/ Healthcare | Responsive alcohol serving training and law enforcement in licenced premises | Do nothing | RCT, CUA, CBA | 12/  NR | QALY, legal costs, productivity losses, health care costs |  |
| 19. | Rehm et al (2011) [19] | Canada/ Societal | Increase taxes | Current Practice | CEA | 60/  3% | Life years, Medical and productivity costs |  |
|  |  |  | Reduced BAC 8% to 5% |  |  |  |  |  |
|  |  |  | Zero BAC for ≤21 years of age |  |  |  |  |  |
|  |  |  | Raising legal age for alcohol consumption |  |  |  |  |  |
|  |  |  | Reduction in crime in bars by 15% |  |  |  |  |  |
|  |  |  | Brief Intervention |  |  |  |  |  |
|  |  |  | Privatisation |  |  |  |  |  |
| 20. | Cobiac et al (2009) [20] | Australia/ Societal | Alcohol screening by random breath-analyser testing | Current Practice | CUA | 12/  NR | DALY |  |
|  |  |  | Raising legal age for alcohol consumption | Current Practice |  |  |  |  |
|  |  |  | Road Safety Campaigns Addressing Drink Driving | Current Practice |  |  |  |  |
|  |  |  | Brief Intervention | Current Practice |  |  |  |  |
|  |  |  | Volumetric Taxation | Current Practice |  |  |  |  |
|  |  |  | Residential Treatment for Alcohol Dependence | Current Practice |  |  |  |  |
| 21. | Chisholm et al (2018) [21] | Global/ Healthcare | Increased Excise Taxation on Alcohol Beverages | Current Practice | CEA | Lifetime/ 3% | Life Years |  |
|  |  |  | Advertisement bans |  |  |  |  |  |
|  |  |  | Reduced Hours of Sale |  |  |  |  |  |
|  |  |  | Strengthening law enforcement regulating alcohol-impaired driving and BAC thresholds |  |  |  |  |  |
|  |  |  | Brief interventions targeting hazardous and harmful patterns of alcohol use |  |  |  |  |  |
| 22. | Brennan et al (2014) [22] | United Kingdom/ Societal | Below-Cost Selling Bans | Minimum Unit Pricing | CUA | Lifetime/  3% | Alcohol consumption, Alcohol related events, Change in mortality rate, QALY |  |
| 23. | Chisholm et al (2004) [23] | Global/ Healthcare | Brief Intervention | Current Practice | CUA | 120/ 3.5% | DALY |  |
|  |  |  | Law Enforcement Activities Including Random Alcohol Screening of Drivers |  |  |  |  |  |
|  |  |  | Taxes on Alcohol Sales |  |  |  |  |  |
|  |  |  | Strengthening law enforcement regulating alcohol-impaired driving |  |  |  |  |  |
|  |  |  | Limitations on the Number and Operation of Licensed Alcohol Outlets |  |  |  |  |  |
|  |  |  | Advertisement Bans |  |  |  |  |  |
|  |  |  | Media-Based Awareness Initiatives |  |  |  |  |  |
| 24. | Purshouse et al (2010) [24] | United Kingdom/ Societal | Per unit alcohol price of £0.70 | Current Practice | CUA | Lifetime/  3% | QALY |  |
|  |  |  | Per unit alcohol price of £0.50 |  |  |  |  |  |
|  |  |  | Per unit alcohol price of £0.4 (Off-Trade) and £1.0 (On-Trade) |  |  |  |  |  |
|  |  |  | Per unit alcohol price of £0.45 |  |  |  |  |  |
|  |  |  | 10% increase in price |  |  |  |  |  |
|  |  |  | Removal of Off-Trade Discounts |  |  |  |  |  |
|  |  |  | Per unit alcohol price of £0.4 |  |  |  |  |  |
|  |  |  | Per unit alcohol price of £0.3 (Off-Trade) and £0.8 (On-Trade) |  |  |  |  |  |
|  |  |  | Per unit alcohol price of £0.30 |  |  |  |  |  |
|  |  |  | 10% price increase for economically priced alcohol |  |  |  |  |  |
|  |  |  | Ban on Price Reductions Over 10% for Alcohol Sold in Off-Trade Outlets |  |  |  |  |  |
|  |  |  | Minimum Price of £0.20 Per Unit of Alcohol |  |  |  |  |  |
|  |  |  | Ban on Price Reductions Over 20% for Alcohol Sold in Off-Trade Outlets |  |  |  |  |  |
|  |  |  | Ban on Price Reductions Over 30% for Alcohol Sold in Off-Trade Outlets |  |  |  |  |  |
|  |  |  | Ban on Price Reductions Over 40% for Alcohol Sold in Off-Trade Outlets |  |  |  |  |  |
|  |  |  | Ban on Price Reductions Over 50% for Alcohol Sold in Off-Trade Outlets |  |  |  |  |  |
| 25. | Henke et al (2011) [25] | USA/ Societal | Health promotion programme at workplace | Do nothing | CBA | 120/ 3.50% | Health care costs |  |
| 26. | Ingels et al (2013) [26] | USA/ Societal | Skill training programme for African American families | Attention Control Intervention | CEA | 72/  NR | Alcohol consumption |  |
| 27. | Kuklinski et al (2015) [27] | USA/ Societal | Community mobilization for prevention of alcohol use | No intervention | RCT, CBA | 18/  NR | Monetary valuation of sustained abstinence from delinquency, alcohol use, and tobacco use |  |
| 28. | Long et al (1998) [28] | United Kingdom/ Healthcare | 2 Week in And Day Patient Treatment | 5-Week Residential Program | RCT, CEA | 60/  3% | Absenteeism rate, Alcohol consumption |  |
| 29. | Miller et al (2008) [29] | USA/ Societal and Healthcare | School Based Interventions to Prevent Substance Abuse | No Direct Comparator | CBA | Lifetime/ 3% | Medical and Productivity Costs |  |
| 30. | Nalpas et al (2003) [30] | France/ Societal and Healthcare | Alcohol Treatment Programme | Do Nothing | CEA | 12/  NR | Relapse of patients |  |
| 31. | Schramm et al (1977) [31] | USA/ Health sector | Employee Health Programme | Do Nothing | CEA | 36/  NR | Reduced absenteeism |  |
| 32. | Spoth et al (2002) [32] | USA/ Healthcare | Skill training for families (parents and children) of school children (7 sessions) | Do Nothing | CEA, CBA | 12/  NR | Cases of youth alcohol use prevented. Monetary valuation of health outcome |  |
|  |  |  | Parenting Skill training (5 sessions) |  |  |  |  |  |
|  |  |  | Health Information |  |  |  |  |  |
| 33. | Tanaree et al (2019) [33] | Thailand/ Societal | Integrated alcohol management in health system | Do Nothing | RCT, CEA | Lifetime/  3% | Treatment uptake |  |
| 34. | Thanh et al (2015) [34] | Canada/ Societal | Three-year home visits for prevention of births exposed to alcohol | No Intervention Scenario | CEA, CBA | 60/  3% | Prevented Fatal Alcohol Spectrum Disorders (FSAD), lifetime cost per case with FASD |  |
| 35. | Angus et al (2014) [35] | Italy/ Societal | SBI at Next GP Registration | Do Nothing | CUA | 36/  5% | QALY |  |
|  |  |  | SBI at Next GP Consultation | Do Nothing |  |  |  |  |
| 36. | Angus et al (2014) [35] | Italy/ Healthcare | SBI | No Intervention | CUA | 120/  3.5% | QALY |  |
| 37. | Angus et al (2017) [36] | Europe/ Healthcare | SBI | Do Nothing | CUA | 360/  3% | QALY |  |
| 38. | Tariq, et al (2009) [37] | Netherlands/ Healthcare | SBI | Usual Care | CUA | 360/  3% | QALY |  |
| 39. | Gentilello et al (2005) [38] | USA/ Healthcare | SBI | No Brief Intervention and Screening | CEA | Lifetime/ 4% | Treatment uptake |  |
| 40. | Havard et al (2012) [39] | Australia/ Healthcare | Brief Intervention during emergency department visit followed by personalised feedback through e-mail | No Further Contact | CEA | 720/  3% | Alcohol consumption with alcohol related events |  |
| 41. | Kunz et al (2004) [40] | USA/ Healthcare | SBI | Control | RCT, CEA | 1.5/  NR | Alcohol consumption |  |
| 42. | Mundt et al (2006) [41] | USA/ Societal | SBI | Control Group | RCT, CEA | 3/  NR | Alcohol related events |  |
| 43. | Navarro et al (2010) [42] | Australia/ Societal | SBI | Control Group | RCT, CEA | 48/  NR | Alcohol consumption |  |
| 44. | Paltzer et al (2019) [43] | USA/ Societal | SBIRT | Usual Care | CEA | 12/  NR | Treatment uptake |  |
| 45. | Pringle et al (2018) [44] | USA/ Healthcare | SBIRT during emergency department admission | Usual Emergency Department Services | RCT, CEA | 24/  NR | Treatment uptake |  |
| 46. | Purshouse et al (2013) [45] | United Kingdom/ Healthcare | SBI | Usual Care | CUA | 12/  NR | QALY |  |
| 47. | Quanbeck et al (2010) [46] | USA/ Healthcare | SBIRT | Do Nothing | CEA | 360/  3.50% | Reduced absenteeism |  |
| 48. | Shanahan et al (2006) [47] | Australia/ Payer | Academic Detailing | Current Practice | CEA | 48/  3.50% | Alcohol consumption, Life Years |  |
|  |  |  | Collaborative Continuing Education for Medical Professionals | Current Practice |  |  |  |  |
|  |  |  | Computerised Reminders | Current Practice |  |  |  |  |
|  |  |  | Targeted Payments | Current Practice |  |  |  |  |
| 49. | Tariq et al (2009) [37] | Netherlands/ Societal | Opportunistic SBI | Current Scenario (No SBI) | CUA | Lifetime/  3% | QALY, LY |  |
| 50. | Zur and Zaric (2016) [48] | Canada/ Healthcare | SBI | No SBI (Individual) | CUA | Lifetime/  4% | QALY, LY |  |
| 51. | Babor et al (2006) [49] | USA/ Healthcare | Brief Intervention by licensed practitioners | Usual care | RCT, CUA | 12/  NR | Alcohol consumption, QALY |  |
|  |  |  | Brief Intervention by nurses | Usual care |  |  |  |  |
| 52. | Barbosa et al (2015) [50] | USA/ Societal | SBIRT | No SBI | CUA | 12/  NR | Alcohol consumption, QALY |  |
| 53. | Smit et al (2011) [51] | Netherlands/ Healthcare | Adding E-health Systems to Conventional Care | Current Practice (No E-health Intervention) | CUA, CBA | 12/  NR | Alcohol consumption, DALY, treatment cost |  |
|  |  |  | Face-To-Face with E-health Interventions by 50% | Current Practice (No E-health Intervention) |  |  |  |  |
| 54. | Lock et al (2006) [52] | USA/ Societal | Nurse-Led Screening and Brief Intervention | Standard Management | RCT, CEA | 12/  NR | Alcohol consumption |  |
| 55. | Kapoor et al (2009) [53] | USA/ Societal | CDT along with questionnaire | CDT Testing | CUA | Lifetime/ 3% | QALY |  |
| 56. | Miller et al (2007) [54] | USA/ Healthcare | The Peer care With Drug Testing | Current Practice | CBA | Lifetime/ 3% | Alcohol related events, employer costs for treatment of injuries |  |
| 57. | Cowell et al (2012) [55] | USA/ Healthcare | Assessment Only | Intergroup Comparison | RCT, CEA | 12/  NR | Alcohol consumption |  |
|  |  |  | Motivational Interviewing |  |  |  |  |  |
|  |  |  | Feedback Only |  |  |  |  |  |
|  |  |  | MIFB (Motivational Interviewing and Feedback) |  |  |  |  |  |
| 58. | Deluca et al (2021) [56] | United Kingdom/ Societal | Screening | Do Nothing | RCT, CUA | 12/  NR | QALY |  |
|  |  |  | PFBA Alongside a Web- or Smartphone-Enabled Electronic Brief Intervention |  |  |  |  |  |
|  |  |  | In-Person Session of Personalized Feedback-Based Assessment |  |  |  |  |  |
| 59. | Downs and Klein (1995) [57] | USA/ Societal | Preventive Health Screenings for Teenagers and Targeted Counselling Sessions for At-Risk Youth | Standard Practice | RCT, CEA | 4/  NR | Alcohol consumption |  |
| 60. | Watson J et al (2013) [58] | United Kingdom/ Societal | Stepped Care with opportunistic screening in Primary Care | Brief Advice by Nurse | RCT, CUA | 12/  NR | Alcohol consumption, QALY |  |
| 61. | Byrnes et al (2010) [59] | Australia/ Healthcare | Alcohol Taxes Based on Volume Without Increasing Deadweight Loss | Current Practice | CUA | 240/  3% | Alcohol consumption, Tax revenue, DALY |  |
|  |  |  | No Alteration in the Taxation Rate | Current Practice |  |  |  |  |
|  |  |  | Uniform Tax Rate Applied to All Alcohol Beverages Equivalent to That for Spirits | Current Practice |  |  |  |  |
| 62. | van den Berg et al (2008) [60] | Netherlands/ Healthcare | Increasing alcohol tax | Current Practice | CUA | Lifetime/ 4% | QALY |  |
| 63. | Cobiac et al (2018) [61] | New Zealand/ Societal | Increase in taxation on alcohol | Current Practice | CUA | Lifetime/ 3% | QALY |  |
| 64. | Doran et al (2013) [62] | Australia/ Healthcare | Replacing wine equalisation tax with a volumetric tax | Current Practice | CUA | Lifetime/  3.5% | Alcohol consumption, DALY |  |
|  |  |  | 10% increase in excise tax on spirits and other beverages |  |  |  |  |  |
|  |  |  | Two-tiered volumetric tax |  |  |  |  |  |
|  |  |  | Exponential increase in excise tax rate by 3% for every 1% increment in alcohol concentration exceeding 3.2% |  |  |  |  |  |
| 65. | Holm et al (2014) [63] | Denmark/ Healthcare | 20% Surge in Taxation Levels | Do Nothing | CUA | Lifetime/  3% | DALY |  |
|  |  |  | Twofold Rise in Taxation |  |  |  |  |  |
|  |  |  | 10% Surge in Taxation Levels |  |  |  |  |  |
| 66. | Holm et al (2014) [64] | Denmark/ Healthcare | Increase in taxation by 30% | Do Nothing | CUA | Lifetime/  3% | DALY |  |
|  |  |  | Advertising Bans on alcohol |  |  |  |  |  |
|  |  |  | Restricted Operating Hours for Alcohol Retail Sales |  |  |  |  |  |
|  |  |  | Telephone based brief Interventions |  |  |  |  |  |
|  |  |  | Extended Intervention Services Provided at Municipal Prevention Centres |  |  |  |  |  |
|  |  |  | Increased Minimum Legal Drinking Age |  |  |  |  |  |
| 67. | Patra et al (2011) [65] | Canada/ Societal | Tax Increase | Current Practice | CEA | 12/  NR | Alcohol related events |  |
|  |  |  | Reducing BAC threshold from 0.08% to 0.05% |  |  |  |  |  |
|  |  |  | Zero BAC threshold for ≤21 years of age |  |  |  |  |  |
|  |  |  | Raiding the legal age for drinking to 21 years |  |  |  |  |  |
|  |  |  | Promotion of safer bars |  |  |  |  |  |
|  |  |  | Privatisation of alcohol sales |  |  |  |  |  |
|  |  |  | Brief Interventions |  |  |  |  |  |
| 68. | van den Berg et al (2008) [60] | Netherlands/ Healthcare | Increasing Alcohol Tax (Dutch Scenario) | Current Practice | CUA | Lifetime/ 4% | QALY |  |
|  |  |  | Increasing Alcohol Tax (Swedish Scenario) |  |  |  |  |  |
| 69. | Blankers et al (2012) [66] | Netherlands/ Societal | Internet-Based Intervention | Internet-Based Self-Help | RCT, CUA | 6/  NR | QALY |  |
| 70. | Brodtkorb et al (2016) [67] | United Kingdom/ Societal | Psychosocial Support with nalmefene | Psychosocial Support | RCT, CUA | 60/  3.5% | QALY |  |
| 71. | Coulton et al (2017) [68] | United Kingdom/ Societal | Stepped Care Intervention: 20 Minute-counselling, 3 sessions of Motivational Enhancement Therapy and referral | Comparator: 5 minutes of brief advice | RCT, CUA | 12/  NR | Alcohol consumption, Alcohol related events, QALY |  |
| 72. | Cowell et al (2018) [69] | USA/ Probation system | Computerised Intervention, Motivational Assessment Program | Face-To-Face Motivational Interviewing | RCT, CEA | 12/ NR | Treatment uptake |  |
| 73. | Crawford et al (2015) [70] | United Kingdom/ Societal | Brief Advice and feedback, appointment offer with health worker | Control Group- written information on health and good lifestyle | RCT, CUA | 6/ NR | Alcohol consumption, QALY |  |
| 74. | Ditsuwan et al (2013) [71] | Thailand/ Healthcare | Psychological Intervention | Do Nothing | CUA | Lifetime/  3% | Alcohol related events, DALY |  |
| 75. | Drummond et al (2009) [72] | United Kingdom/ Societal | Stepped Care Alcohol Intervention | Brief Intervention | RCT, CUA | 6/  NR | Alcohol consumption, Alcohol related events, QALY |  |
| 76. | Dunlap et al (2010) [73] | USA/ Societal | Medical Management  (MM) | Intergroup Comparison | RCT, CEA | 4/  NR | Alcohol abstinence |  |
|  |  |  | Medical management along with Naltrexone |  |  |  |  |  |
|  |  |  | Medical management along with Naltrexone and Acamprosate |  |  |  |  |  |
|  |  |  | Combined Behavioural Intervention |  |  |  |  |  |
|  |  |  | Medical management along with Acamprosate |  |  |  |  |  |
|  |  |  | Medical management along with Placebo and Combined Behavioural Intervention |  |  |  |  |  |
|  |  |  | Medical management along with Naltrexone and Combined Behavioural Intervention |  |  |  |  |  |
|  |  |  | Medical management along with Naltrexone, Acamprosate and Combined Behavioural Intervention |  |  |  |  |  |
|  |  |  | Medical management along with Acamprosate and Combined Behavioural Intervention |  |  |  |  |  |
| 77. | Galárraga et al (2017) [74] | Kenya/ Societal | CBT by Paraprofessionals | CBT by Mental Health Professionals | CEA | 72/  3% | HIV incidence, Labor force and Household productivity |  |
| 78. | Kruger et al (2014) [75] | United Kingdom/ Healthcare | Online Health Behaviour Intervention | Current Practice | RCT, CUA | 6/  1.50% | QALY, LY |  |
| 79. | Lai et al (2007) [76] | Estonia/ Healthcare | Increased Taxation (25%) | Current Practice | CUA | Lifetime/ 3% | DALY |  |
|  |  |  | Increased Taxation (50%) |  |  |  |  |  |
|  |  |  | Alcohol Breath-Testing |  |  |  |  |  |
|  |  |  | Restricted Availability of Alcohol Through Retail Channels |  |  |  |  |  |
|  |  |  | Advertising Bans for alcohol |  |  |  |  |  |
|  |  |  | Brief Advice in Primary Care |  |  |  |  |  |
| 80. | Laramee et al (2016) [77] | United Kingdom/ Healthcare | Psychosocial Support with nalmefene | Psychosocial Support | CUA | 60/ 3.5% | QALY |  |
| 81. | Laramee et al (2014) [78] | United Kingdom/ Healthcare | Psychosocial Support with nalmefene | Psychosocial Support | CUA | 60/  3.5% | Alcohol related events, QALY |  |
| 82. | Millier et al (2017) [79] | United Kingdom/ Societal | Psychosocial Support with nalmefene | Psychosocial Support | CUA | 60/ 3.5% | QALY |  |
| 83. | Mortimer et al (2005) [80] | Australia/ Societal | Targeted Brief Intervention Strategies for Problematic Alcohol Use | No Alcohol-Related Treatment | CUA | Lifetime/ 5% | QALY |  |
|  |  |  | Psychotherapy for mild to moderate alcohol dependency | Behavioural Self-Control Training (BSCT) |  |  |  |  |
|  |  |  | Motivational enhancement therapy for mild to moderate dependence | No intervention |  |  |  |  |
|  |  |  | Naltrexone + Counselling (Severe Dependence) | Placebo + Counselling |  |  |  |  |
| 84. | Neighbors et al (2010) [81] | USA/ Societal | Motivational Interviewing | Standard care (brief advice) | CEA | 6/  NR | Alcohol related events |  |
| 85. | O’Farrell et al (1996) [82] | USA/ Societal | Individual Alcoholism Counselling, | Intergroup Comparison | RCT, CEA, CBA | 24/  NR | Alcohol consumption, Healthcare and legal costs |  |
|  |  |  | Behavioural Marital Therapy |  |  |  |  |  |
|  |  |  | Interactive Couples Group |  |  |  |  |  |
| 86. | Palmer et al. (2000) [83] | Germany/ Healthcare | Counselling with adjuvant Acamprosate for 48 weeks | Counselling | CEA | Lifetime/ 5% | Life years |  |
| 87. | Parrott et al (2006) [84] | England/ Societal | Smithfield Centre (Manchester): A Direct-Access Alcohol Detoxification Service, Without a Prior Appointment or Referral | Plummer Court (Newcastle): A Structured NHS Partial Hospitalization Program, Which Involved Hospital-Based Care Model | CEA, CUA | 6/ NR | QALY |  |
| 88. | Pettinati et al (1999) [85] | USA/ Societal | 12 Step Program, Inpatient (4 Weeks) | 12 Step Program Outpatient (6weeks) | CEA | 12/  NR | Alcohol related events |  |
| 89. | Rychlik et al (2003) [86] | Germany/ Societal | Adjuvant Acamprosate Therapy | Standard Psychosocial Rehabilitation Programme | CEA | 12/  NR | Alcohol abstinence |  |
| 90. | Schädlich et al (1998) [87] | Germany/ Healthcare | Adjuvant Acamprosate Therapy | Standard Psychosocial Rehabilitation Programme | Model based analysis, CEA | 120/  NR | Alcohol abstinence |  |
| 91. | Schulz et al (2014) [88] | Netherlands/ Societal | Sequential Condition | Control (Minimal Intervention) | RCT, CUA | 24/  4% | QALY |  |
|  |  |  | Simultaneous Condition | Control (Minimal Intervention) |  |  |  |  |
| 92. | Sluiter et al. (2018) [89] | Netherlands/ Societal | Genotype-Guided Treatment | Standard Care (Random Treatment Allocation to Acamprosate or Naltrexone) | CUA | 12/  NR | QALY |  |
| 93. | UKATT Research Team. (2005) [90] | United Kingdom/ Societal | Social Behavioural and Network Therapy | Motivational Enhancement Therapy | RCT, CUA | 12/  NR | QALY |  |
| 94. | Walters et al (2009) [91] | Australia/ Healthcare | CBT + Naltrexone | CBT Alone | CEA, CUA | 3/  NR | Alcohol abstinence, QALY |  |
| 95. | Watson et al (2015) [92] | United Kingdom/ Societal | Counselling session focussed on Healthy Living Interventions | Support for behaviour change in problem drinkers | RCT, CUA | 12/  NR | Alcohol Consumption, QALY |  |
| 96. | Zarkin et al (2008) [93] | USA/ Healthcare | Medical Management along with Placebo | Intergroup Comparison | RCT, CEA | 4/  NR | Alcohol consumption. Alcohol abstinence |  |
|  |  |  | Medical Management along with Naltrexone |  |  |  |  |  |
|  |  |  | Medical Management along with Naltrexone and Acamprosate |  |  |  |  |  |
|  |  |  | Combined Behavioural Intervention |  |  |  |  |  |
|  |  |  | Medical Management along with Acamprosate |  |  |  |  |  |
|  |  |  | Medical Management along with Placebo and Combined Behavioural Intervention |  |  |  |  |  |
|  |  |  | Medical Management along with Naltrexone and Combined Behavioural Intervention |  |  |  |  |  |
|  |  |  | Medical Management along with Naltrexone, Acamprosate and Combined Behavioural Intervention |  |  |  |  |  |
|  |  |  | Medical Management along with Acamprosate and Combined Behavioural Intervention |  |  |  |  |  |
| 97. | Alwyn et al (2004) [94] | United Kingdom/ Healthcare | Psychological Intervention | Universal | RCT, CEA | 12/  NR | Alcohol consumption. Alcohol abstinence |  |
| 98. | Annemans et al (2000) [95] | Belgium/ Healthcare | Acamprosate | Placebo | CEA | 24/  NR | Alcohol abstinence |  |
| 99. | Bischof et al (2008) [96] | Germany/ Healthcare | Stepped Care | Full Care | RCT, CEA | 12/  NR | Alcohol consumption |  |
| 100. | Fals-Stewart et al (2005) [97] | USA/ Societal | Brief Relationship Therapy | Intergroup Comparison | RCT, CEA | 12/  NR | Alcohol consumption |  |
|  |  |  | Shortened Version of Behavioural Therapy |  |  |  |  |  |
|  |  |  | Personalized Treatment Approach |  |  |  |  |  |
|  |  |  | Psychoeducational Therapy with Focused Attention Control |  |  |  |  |  |
| 101. | Shepard et al (2016) [98] | USA/ Societal | Significant Other into Motivational Intervention (SOMI) | Brief Individual Motivational Intervention | RCT, CUA | 12/  NR | Alcohol Consumption, QALY |  |
| 102. | Avancena et al (2021) [99] | USA/ Societal | FDA-Approved Pharmacological Interventions for Alcohol Dependence (Acamprosate and Naltrexone) | Do Nothing | CUA | Lifetime/  3% | QALY |  |
|  |  |  | Non-FDA-Approved Pharmacological Treatments (Baclofen, Topiramate, Gabapentin) Combined with Counselling |  |  |  |  |  |
|  |  |  | Counselling |  |  |  |  |  |

AUDIT: Alcohol Use Disorder Identification Test, AUDIT-C: Alcohol use Disorder Identification test- consumption, BAC: Blood Alcohol Concentration, CBA: Cost benefit analysis, CBT: Cognitive Behavioural Therapy, CDT: Carbohydrate deficient transferrin, CEA: Cost-effectiveness analysis, CUA: Cost utility analysis, DALY: Disability-adjusted life years, FDA: Food and Drug Administration, NR: Not Reported, QALY: Quality-adjusted Life years, RCT: Randomized controlled trial, SBI: Screening and brief intervention, SBIRT: Screening, brief intervention, and referral to treatment, SOMI: Significant other motivational intervention, USA: United States of America

Green boxes represent studies with co-effective strategy conclusion, while red boxes represent not cost-effective strategy conclusion

# **Section 2.** **Background and** Description of screening strategy

The Mental, Neurological, and Substance Use (MNS) guidelines recommending the screening and management of AUDs were developed by the Ministry of Health and Family Welfare (MoHFW), Government of India, as part of the expanded primary healthcare programme. However, since health is a state subject in India, the implementation of centrally formulated programmes and policies rests with individual state governments. Accordingly, this analysis was undertaken to develop an investment case to inform the decision-making for the implementation of services for early detection and management of alcohol use disorders. This economic analysis of the policy was also commissioned by the Health Technology Assessment agency within the Ministry of Health and Family Welfare of India to generate economic evidence for efficient resource allocation and strengthen advocacy for policy implementation.

The national MNS guidelines provide flexibility to states regarding the age groups eligible for screening. Based on consultations with clinical experts and national-level policymakers, the primary analysis was conducted for the 15–74 years age group. In addition, a scenario analysis was undertaken for the 30–74 years age group, consistent with the target population for most non-communicable disease screening programmes in India, to ensure policy comparability and relevance.

Based on the mental, neurological, and substance use disorders’ (MNS) guidelines of comprehensive primary healthcare in India, it was considered that the community health officers (CHOs) [community health worker with qualification of a nurse or doctor of traditional, complementary or alternative medicine, who are in charge of the first point of contact government primary healthcare facilities] would perform face-to-face screening of AUDs at the sub-centre- AAMs (first point of facility contact for primary healthcare), using the Alcohol Use Disorder Identification Test (AUDIT) tool (Figure S1) [100]. In the primary analysis, it was assumed that annual screening would be conducted in 15-74 years aged cohort on annual basis and was modelled as a proactive, population-based strategy, rather than an opportunistic or passive approach restricted to individuals presenting to health facilities. The timeframe for implementation for the purpose of this economic analysis was restricted to 10 years, taking into account the anticipated shifts in healthcare systems, and the healthcare-seeking behaviour beyond the period in India.

Figure S1. Comparative scenarios

^AUD: Alcohol Use Disorder, CHO: Community Health Officer, ASHA: Accredited Social Health Activist, AUDIT: Alcohol Use Disorder Identification Test, LFT: Liver Function Test, RFT: Renal Function Test^

Based on the expert consultation, it was assumed that a capacity-building session would be organized at the district level, led by a psychiatrist and the program implementation team, to equip the CHOs with the necessary skills to carry out the universal screening program. Subsequently, the CHOs would train the Accredited Social Health Activists (ASHAs) [community health worker who is the point of contact between community and government health facilities] to collaboratively implement the program, as, it is anticipated that ASHAs would play a crucial role in ongoing social mobilization efforts to foster community participation in the screening initiative. Furthermore, it was considered that medical officers would be involved in supportive supervision of the screening programme.

# **Section 3**. Methods for costing of screening services for alcohol use disorders

A normative costing approach was employed to estimate the implementation costs for screening of alcohol use disorders (AUDs) at *Ayushman Arogya Mandirs* (AAM) - sub-centre, utilizing empirical cost data from primary healthcare studies archived in the National Health System Cost Database of India.

In accordance with national operational guidelines, it was assumed that Community Health Officers (CHOs) would conduct screening for AUDs using the Alcohol Use Disorders Identification Test (AUDIT), targeting either the 15–74 years or 30–74 years age group. In addition to conducting the assessments, CHOs and Accredited Social Health Activists (ASHAs) would also undertake social mobilization activities to enhance community participation and awareness regarding the screening services at AAM sub-centres.

The implementation costs included salaries of human resources, apportioned based on the time spent on screening-related activities; costs of stationery; information, education, and communication (IEC) materials; and utility expenses (electricity and water) incurred during screening days. Data on the economic valuation of physical space, utility charges, and other related cost inputs were sourced from existing Indian primary healthcare costing studies within the National Health System Cost Database.^[1]^ Staff salaries were similarly drawn from this database. All cost estimates from earlier studies were adjusted to 2022 price levels using GDP deflator values. Capital and non-capital resources were apportioned based on a factor reflecting the anticipated number of individuals to be screened from the catchment population (based on service coverage rates) and the total patient footfall at each sub-centre, with data drawn from the National Health System Cost Database.

In addition to implementation costs, expenditures related to training and supportive supervision were incorporated. Based on expert consultations, it was assumed that a one-day capacity-building programme would be organized for CHOs and ASHAs, facilitated by a psychiatrist and a programme manager, to equip staff with competencies in administering the AUDIT tool, providing initial management, and conducting community mobilization activities. The cost components for training, including rental charges for a training venue at the district hospital, salaries of trainers, costs of furniture, and training materials were sourced from the National Health System Cost Database. Incentive payments to CHOs and ASHAs for participating in training were estimated based on financial data obtained through consultations with state government officials in northern India. Training-related resource use was apportioned based on time spent, and training costs were annualized over a five-year period using a 3% annual discount rate. Under the assumption of a centralized training model, wherein staff from all sub-centres within a district would be trained in a single session at a district-level facility, the per-person training cost was derived accordingly.

Supportive supervision was also included in the costing framework. It was assumed that each sub-centre would receive six supervisory visits annually by designated health authorities. The associated costs included salaries of supervising officials, CHOs, and ASHAs, as well as costs of travel, space, furniture, and stationery used during supervisory activities.

To estimate the screening programme's outputs, population coverage rates were applied to determine the number of individuals expected to undergo AUDIT screening. Given that each SC typically serves a catchment population of approximately 5,000 individuals, and that 72% and 45% of this population fall within the 15–74 years and 30–74 years age groups, respectively, the eligible population for screening was calculated accordingly. The number of individuals expected to be screened using the AUDIT tool was then estimated across five different coverage scenarios: 50%, 60%, 70%, 80%, and 90%.

Table S2. Per person cost of AUDIT based screening according to variations in screening coverages (₹)

| Screening Coverage Rate | AUDIT screening  (Age group: 15-74 years old) | AUDIT screening  (Age group: 30 years-74 years old) |
| --- | --- | --- |
| 50% | 30.07 | 30.90 |
| 60% | 29.79 | 30.56 |
| 70% | 29.55 | 30.30 |
| 80% | 29.35 | 30.08 |
| 90% | 29.18 | 29.90 |

AUDIT: Alcohol Use Disorder Identification Test

## Break-up of costs for implementation of alcohol use disorder screening programme

Human resources and capital costs contributed to the majority of the health systems costs to implement depression screening programme (Figure S1). Human resources had a share of 79% in the overall costs and equipment contributed to 8% of the costs.

Figure S2. Details of cost components in screening scenario

|  |  |
| --- | --- |
| Human Resource | 58764.4 |
| Equipment | 0 |
| Capital | 6321.23 |
| Furniture | 3483.08 |
| Overheads | 2515.17 |
| Stationary | 984.36 |
| IEC | 125 |
| Training and supervision | 2524 |

IEC: Information, Education, and Communication material

# **Section 4**. Model related structures and parameters

## **Section 4A. Decision Tree Analysis**

Table S3. Base value of parameters for decision tree analysis and data sources

| Parameters | Base value | Standard Error | Distribution | Source |
| --- | --- | --- | --- | --- |
| Prevalence of drinking in 12 months (Males) |  |  |  |  |
| 15-19 years | 0.08 | 0.004 | Beta | [101] |
| 20-24 years | 0.22 | 0.013 |  |  |
| 25-29 years | 0.32 | 0.018 |  |  |
| 30-34 years | 0.38 | 0.022 |  |  |
| 35-39 years | 0.38 | 0.022 |  |  |
| 40-44 years | 0.39 | 0.022 |  |  |
| 45-49 years | 0.36 | 0.021 |  |  |
| 50-54 years | 0.35 | 0.020 |  |  |
| 55-59 years | 0.34 | 0.019 |  |  |
| 60-64 years | 0.32 | 0.018 |  |  |
| 65-69 years | 0.30 | 0.017 |  |  |
| 70-74 years | 0.27 | 0.015 |  |  |
| Prevalence of drinking in 12 months (Females) |  |  |  |  |
| 15-19 years | 0.003 | 0.0002 | Beta | [101] |
| 20-24 years | 0.01 | 0.0005 |  |  |
| 25-29 years | 0.01 | 0.0008 |  |  |
| 30-34 years | 0.04 | 0.0023 |  |  |
| 35-39 years | 0.02 | 0.0012 |  |  |
| 40-44 years | 0.02 | 0.0013 |  |  |
| 45-49 years | 0.02 | 0.0010 |  |  |
| 50-54 years | 0.02 | 0.0010 |  |  |
| 55-59 years | 0.02 | 0.0009 |  |  |
| 60-64 years | 0.02 | 0.0009 |  |  |
| 65-69 years | 0.02 | 0.0010 |  |  |
| 70-74 years | 0.01 | 0.0006 |  |  |
| Proportion of alcohol consumptions (Males) |  |  |  |  |
| Low risk use (Males) |  |  |  |  |
| 15-19 years | 0.42 | 0.0242 | Beta | [101] |
| 20-24 years | 0.37 | 0.0214 |  |  |
| 25-29 years | 0.38 | 0.0219 |  |  |
| 30-34 years | 0.33 | 0.0191 |  |  |
| 35-39 years | 0.35 | 0.0202 |  |  |
| 40-44 years | 0.36 | 0.0208 |  |  |
| 45-49 years | 0.35 | 0.0202 |  |  |
| 50-54 years | 0.35 | 0.0202 |  |  |
| 55-59 years | 0.37 | 0.0214 |  |  |
| 60-64 years | 0.33 | 0.0191 |  |  |
| 65-69 years | 0.39 | 0.0225 |  |  |
| 70-74 years | 0.34 | 0.0196 |  |  |
| Hazardous use (Males) |  |  |  |  |
| 15-19 years | 0.32 | 0.0185 | Beta | [101] |
| 20-24 years | 0.29 | 0.0167 |  |  |
| 25-29 years | 0.29 | 0.0167 |  |  |
| 30-34 years | 0.25 | 0.0144 |  |  |
| 35-39 years | 0.27 | 0.0156 |  |  |
| 40-44 years | 0.27 | 0.0156 |  |  |
| 45-49 years | 0.27 | 0.0156 |  |  |
| 50-54 years | 0.27 | 0.0156 |  |  |
| 55-59 years | 0.28 | 0.0162 |  |  |
| 60-64 years | 0.25 | 0.0144 |  |  |
| 65-69 years | 0.30 | 0.0173 |  |  |
| 70-74 years | 0.26 | 0.0150 |  |  |
| Harmful use (Males) |  |  |  |  |
| 15-19 years | 0.16 | 0.0092 | Beta | [101] |
| 20-24 years | 0.22 | 0.0127 |  |  |
| 25-29 years | 0.15 | 0.0087 |  |  |
| 30-34 years | 0.15 | 0.0087 |  |  |
| 35-39 years | 0.20 | 0.0115 |  |  |
| 40-44 years | 0.17 | 0.0098 |  |  |
| 45-49 years | 0.16 | 0.0092 |  |  |
| 50-54 years | 0.19 | 0.0110 |  |  |
| 55-59 years | 0.14 | 0.0081 |  |  |
| 60-64 years | 0.26 | 0.0150 |  |  |
| 65-69 years | 0.13 | 0.0075 |  |  |
| 70-74 years | 0.14 | 0.0081 |  |  |
| Alcohol dependence (Males) |  |  |  |  |
| 15-19 years | 0.09 | 0.0052 | Beta | [101] |
| 20-24 years | 0.12 | 0.0069 |  |  |
| 25-29 years | 0.18 | 0.0104 |  |  |
| 30-34 years | 0.27 | 0.0156 |  |  |
| 35-39 years | 0.18 | 0.0104 |  |  |
| 40-44 years | 0.20 | 0.0115 |  |  |
| 45-49 years | 0.22 | 0.0127 |  |  |
| 50-54 years | 0.19 | 0.0110 |  |  |
| 55-59 years | 0.21 | 0.0121 |  |  |
| 60-64 years | 0.16 | 0.0092 |  |  |
| 65-69 years | 0.18 | 0.0104 |  |  |
| 70-74 years | 0.26 | 0.0150 |  |  |
| Proportion of alcohol consumptions (Females) |  |  |  |  |
| Low risk use (Females) |  |  |  |  |
| 15-19 years | 0.50 | 0.0289 | Beta | [101] |
| 20-24 years | 0.49 | 0.0283 |  |  |
| 25-29 years | 0.40 | 0.0231 |  |  |
| 30-34 years | 0.53 | 0.0306 |  |  |
| 35-39 years | 0.52 | 0.0300 |  |  |
| 40-44 years | 0.43 | 0.0248 |  |  |
| 45-49 years | 0.48 | 0.0277 |  |  |
| 50-54 years | 0.42 | 0.0242 |  |  |
| 55-59 years | 0.51 | 0.0294 |  |  |
| 60-64 years | 0.48 | 0.0277 |  |  |
| 65-69 years | 0.43 | 0.0248 |  |  |
| 70-74 years | 0.49 | 0.0283 |  |  |
| Hazardous use (Females) |  |  |  |  |
| 15-19 years | 0.38 | 0.0219 | Beta | [101] |
| 20-24 years | 0.37 | 0.0214 |  |  |
| 25-29 years | 0.31 | 0.0179 |  |  |
| 30-34 years | 0.40 | 0.0231 |  |  |
| 35-39 years | 0.39 | 0.0225 |  |  |
| 40-44 years | 0.33 | 0.0191 |  |  |
| 45-49 years | 0.37 | 0.0214 |  |  |
| 50-54 years | 0.32 | 0.0185 |  |  |
| 55-59 years | 0.39 | 0.0225 |  |  |
| 60-64 years | 0.37 | 0.0214 |  |  |
| 65-69 years | 0.33 | 0.0191 |  |  |
| 70-74 years | 0.38 | 0.0219 |  |  |
| Harmful use (Females) |  |  |  |  |
| 15-19 years | 0.05 | 0.0029 | Beta | [101] |
| 20-24 years | 0.08 | 0.0046 |  |  |
| 25-29 years | 0.19 | 0.0110 |  |  |
| 30-34 years | 0.04 | 0.0023 |  |  |
| 35-39 years | 0.04 | 0.0023 |  |  |
| 40-44 years | 0.09 | 0.0052 |  |  |
| 45-49 years | 0.09 | 0.0052 |  |  |
| 50-54 years | 0.13 | 0.0075 |  |  |
| 55-59 years | 0.05 | 0.0029 |  |  |
| 60-64 years | 0.08 | 0.0046 |  |  |
| 65-69 years | 0.17 | 0.0098 |  |  |
| 70-74 years | 0.04 | 0.0023 |  |  |
| Alcohol dependence (Females) |  |  |  |  |
| 15-19 years | 0.07 | 0.0040 | Beta | [101] |
| 20-24 years | 0.05 | 0.0029 |  |  |
| 25-29 years | 0.10 | 0.0058 |  |  |
| 30-34 years | 0.03 | 0.0017 |  |  |
| 35-39 years | 0.05 | 0.0029 |  |  |
| 40-44 years | 0.15 | 0.0087 |  |  |
| 45-49 years | 0.06 | 0.0035 |  |  |
| 50-54 years | 0.12 | 0.0069 |  |  |
| 55-59 years | 0.05 | 0.0029 |  |  |
| 60-64 years | 0.08 | 0.0046 |  |  |
| 65-69 years | 0.06 | 0.0035 |  |  |
| 70-74 years | 0.09 | 0.0052 |  |  |
| Screening accuracy (AUDIT score> 8) |  |  |  |  |
| Sensitivity | 0.939 | 0.054 | Normal | [102] |
| Specificity | 0.667 | 0.039 |  |  |
| Coverage rates |  |  |  |  |
| Screening rate | 0.7 | 0.040 | Normal | Expert Consultation |
| Loss to follow-up for diagnosis | 0.15 | 0.009 |  |  |
| Treatment rate (usual care) | 0.137 | 0.008 |  | [103] |
| Treatment rate (screening scenario) | 0.85 | 0.049 |  | Expert Consultation |
| Treatment compliance | 0.7 | 0.040 |  | [104] |
| Care seeking pattern (usual care/ screening scenario) |  |  |  |  |
| *Harmful use* |  |  |  |  |
| SC | 0.03 | 0.002 | Normal | Analysis using [103] |
| PHC | 0.16 | 0.009 |  |  |
| Secondary care | 0.04 | 0.002 |  |  |
| Tertiary care | 0.19 | 0.011 |  |  |
| Private | 0.58 | 0.033 |  |  |
| *Alcohol dependence* |  |  |  |  |
| SC | 0.01 | 0.001 | Normal | Analysis using [103] |
| PHC | 0.18 | 0.104 |  |  |
| Secondary care | 0.08 | 0.005 |  |  |
| Tertiary care | 0.2 | 0.012 |  |  |
| Private | 0.53 | 0.031 |  |  |
| Care seeking pattern (screening scenario) |  |  |  |  |
| *Low risk use* |  |  |  |  |
| SC | 1 |  |  | Expert Consultation |
| *Hazardous use* |  |  |  |  |
| PHC | 0.6 | 0.035 | Normal | Expert Consultation |
| Private | 0.4 | 0.023 |  |  |
| Treatments |  |  |  |  |
| Hazardous use/ Harmful use (Proportion of patients) |  |  |  |  |
| Brief Interventions (15 minutes) | 1 |  | Normal | [105], Expert consultation |
| Alcohol Dependence (Proportion of patients) |  |  |  |  |
| Psychosocial Interventions | 0.5 | 0.028 | Normal | [105], [106], Expert consultation |
| Lorazepam | 0.57 | 0.033 |  |  |
| Chlordiazepoxide | 0.52 | 0.030 |  |  |
| Thiamine | 0.41 | 0.024 |  |  |
| Disulfiram | 0.51 | 0.029 |  |  |
| Acamprosate | 0.45 | 0.026 |  |  |
| Naltrexone | 0.04 | 0.021 |  |  |
| Multivitamins | 0.8 | 0.046 |  |  |
| Treatment of Alcohol Withdrawal Syndrome (Proportion of patients) |  |  |  |  |
| Chlordiazepoxide | 0.48 | 0.028 | Normal | [105]- [107], Expert consultation |
| Lorazepam | 0.68 | 0.039 |  |  |
| Thiamine | 0.03 | 0.001 |  |  |
| Disulfiram | 0.19 | 0.010 |  |  |
| Acamprosate | 0.11 | 0.006 |  |  |
| Naltrexone | 0.02 | 0.001 |  |  |
| Treatment efficacy (Proportion of abstinence following one year of treatment) |  |  |  |  |
| Brief intervention | 0.6 | 0.035 | Beta | [108] |
| Acamprosate | 0.46 | 0.027 |  | [109] |
| Naltrexone | 0.44 | 0.025 |  | [110] |
| Disulfiram | 0.9 | 0.052 |  | [111] |
| Baclofen | 0.7 | 0.040 |  | [112] |
| Annual hospitalization rate | 0.1 | 0.005 |  | Expert Consultation |
| Cost per patient per bed day hospitalization ₹ ($) | 9016  (114) | 521  (7) | Gamma | [113] |
| Number of OPD visits in a year | 12 |  |  | [105] |
| Cost of screening ₹ ($) |  |  |  |  |
| Per person health system cost of screening | 30 (0.50) | 8.661(0.110) | Gamma | Analysis using [114] |
| Per person non-medical cost of screening | 84(1) | 23.671(0.301) |  |  |
| Per diagnostic cost (public) ₹ ($) |  |  |  |  |
| Hemogram | 138 (1.70) | 39.837 (0.506) | Gamma | [105], [115] |
| Liver Function Test | 275(3) | 79.386(1.010) |  |  |
| Renal Function Test | 261(3) | 75.344(0.958) |  |  |
| Ultrasound | 300(4) | 86.603(1.101) |  |  |
| Fibro scan | 1150(14) | 331.976(4.223) |  |  |
| Serum electrolytes | 140(1.80) | 40.415(0.514) |  |  |
| Per diagnostic cost (private) ₹ ($) |  |  |  |  |
| Hemogram | 400(5) | 115.470(1.469) | Gamma | [105], [116-119] |
| Liver Function Test | 370(4.70) | 106.810(1.358) |  |  |
| Renal Function Test | 700(9) | 202.073(2.570) |  |  |
| Ultrasound | 500(6) | 144.338(1.836) |  |  |
| Fibro scan | 6000(76) | 1732.051(22.036) |  |  |
| Serum electrolytes | 350(4) | 101.036(1.285) |  |  |
| Annual Cost of treatment (public) ₹ ($) |  |  |  |  |
| Lorazepam | 193 (3) | 55.686 (0.708) | Gamma | [105], [114] |
| Chlordiazepoxide | 327 (4.60) | 94.408 (1.201) |  |  |
| Thiamine | 90 (1.20) | 26.036 (0.331) |  |  |
| Disulfiram | 456 (6.50) | 131.778 (1.676) |  |  |
| Acamprosate | 6066 (77) | 1751.190 (22.279) |  |  |
| Naltrexone | 7546 (96) | 2178.381 (27.714) |  |  |
| Multivitamins | 165(2) | 47.666 (0.606) |  |  |
| Annual Cost of treatment (private) ₹ ($) |  |  |  |  |
| Lorazepam | 7300 (92) | 2107.328 (26.810) | Gamma | [105], [120] |
| Chlordiazepoxide | 2738 (34) | 790.248 (10.054) |  |  |
| Thiamine | 876 (11) | 252.879 (3.217) |  |  |
| Disulfiram | 1205 (15) | 347.709 (4.423) |  |  |
| Acamprosate | 10402 (132) | 3002.943  (38.205) |  |  |
| Naltrexone | 14400  (183) | 4156.922 (52.887) |  |  |
| Multivitamins | 3600 (45) | 1039.23 (13.221) |  |  |
| Health system Consultation cost per OPD visit ₹ ($) |  |  |  |  |
| PHC | 121 (1.5) | 34.930 (0.444) | Gamma | [104], [114] |
| Government secondary care facility | 186 (2.30) | 53.694 (0.683) |  |  |
| Government tertiary care facility | 990 (12) | 285.788 (3.635) |  |  |
| OOPE per OPD visit₹ ($) |  |  |  |  |
| PHC | 153 (2) | 44.167 (0.561) | Gamma | [104], [121] |
| Government secondary/ tertiary care facility | 195 (2.40) | 56.292 (0.716) |  |  |
| Private | 1500 (19) | 433.013 (5.509) |  |  |
| All-cause mortality (males) |  |  |  |  |
| 15-19 years | 0.00079 | 0.00004 | Beta | [122] |
| 20-24 years | 0.00124 | 0.00007 |  |  |
| 25-29 years | 0.00155 | 0.00008 |  |  |
| 30-34 years | 0.00214 | 0.00012 |  |  |
| 35-39 years | 0.00299 | 0.00017 |  |  |
| 40-44 years | 0.00442 | 0.00026 |  |  |
| 45-49 years | 0.00616 | 0.00036 |  |  |
| 50-54 years | 0.00893 | 0.00052 |  |  |
| 55-59 years | 0.01556 | 0.00090 |  |  |
| 60-64 years | 0.02146 | 0.00124 |  |  |
| 65-69 years | 0.03319 | 0.00192 |  |  |
| 70-74 years | 0.05006 | 0.00289 |  |  |
| 75-79 years | 0.07103 | 0.00411 |  |  |
| 80 years and above | 0.10860 | 0.00627 |  |  |
| All-cause mortality (females) |  |  |  |  |
| 15-19 years | 0.00077 | 0.00004 | Beta | [122] |
| 20-24 years | 0.00099 | 0.00005 |  |  |
| 25-29 years | 0.00113 | 0.00006 |  |  |
| 30-34 years | 0.00137 | 0.00007 |  |  |
| 35-39 years | 0.00172 | 0.00009 |  |  |
| 40-44 years | 0.00260 | 0.00015 |  |  |
| 45-49 years | 0.00374 | 0.00022 |  |  |
| 50-54 years | 0.00656 | 0.00038 |  |  |
| 55-59 years | 0.01107 | 0.00064 |  |  |
| 60-64 years | 0.01589 | 0.00092 |  |  |
| 65-69 years | 0.02596 | 0.00150 |  |  |
| 70-74 years | 0.04067 | 0.00235 |  |  |
| 75-79 years | 0.06062 | 0.00350 |  |  |
| 80 years and above | 0.09788 | 0.00565 |  |  |

^AUDIT: Alcohol Use Disorder Identification Test, SC: Sub-centre, PHC: Primary Health Centre, OPD: Outpatient Department, OOPE: Out-of-pocket expenditure, ₹: Indian National Rupee^

## **Section 4B. Markov state transition models**

### *Road traffic Accident Injuries: Model structure and parameters*

The data on road traffic accident injuries (RTAs) was obtained from the most recent report of the Ministry of Road Transport and Highways (2022) [123]. However, considering the under-reporting of RTAs in India, estimated at 85% (Singh et al, 2018) [124], the absolute age specific- number of individuals involved in RTAs was adjusted by a factor of 5.67. Further, age- and gender- specific incidence rates were calculated by dividing the absolute number with the total population in specific age and gender groups. The relative risks of RTAs in low risk, hazardous, harmful, and dependence use, as compared to that in population without alcohol use were obtained from an Indian study (Table 3).

Figure S3. Markov state transition model for Road Traffic Accident Injuries

The road traffic accident injuries were further categorized into mild, moderate, severe injuries (Figure S2), according to the data from Ministry of Road Transport and Highways. In line with the published literature, it was considered that 10%, 50%, and 100% of individuals with mild, moderate, and severe injury, respectively, who survived for one year after the injury, would require long-term rehabilitation, and hence would remain in the same health state [125]. In other words, 90%, and 50% of the individuals with mild and moderate injury would return to ‘healthy’ state in the subsequent cycle. The probability of mortality from RTAs of different severity levels was calculated based on survival rates of patients with varied Glasgow coma scale scores [126]. It was also considered that in a lifetime the individuals could die of causes other than RTAs, and hence, age and gender specific all-cause mortality probabilities were applied, using the life tables from Sample Registration System of India (Figure 3).

The care seeking pattern for RTAs was derived using the National Sample Survey of India, [121]. The health systems costs were derived from the National Health System Cost Database for India, and an Indian study conducted in secondary and tertiary public sector hospitals [127]. The OOPE estimates were based on observational studies conducted in public and private health facilities in India (Table S3). Similarly, utility values reflecting the quality of life of individuals affected by RTIs at different intervals post-injury were sourced from an Indian study [128].

Table S4. Base value of parameters used for markov model on Road traffic accident injuries and data sources

| Parameter | Base value | Standard Error | Distribution | Source |
| --- | --- | --- | --- | --- |
| Incidence of RTA (males) |  |  | Beta |  |
| <18 years | 0.0007 | 0.00004 |  | Analysis using [123] |
| 18-25 years | 0.0059 | 0.00034 |  |  |
| 26-35 years | 0.0068 | 0.00039 |  |  |
| 36-45 years | 0.0072 | 0.00041 |  |  |
| 46-60 years | 0.0050 | 0.00029 |  |  |
| >60 years | 0.0008 | 0.00004 |  |  |
| Incidence of RTA (females) |  |  |  |  |
| <18 years | 0.00018 | 0.00001 | Beta | Analysis using [123] |
| 18-25 years | 0.00073 | 0.00004 |  |  |
| 26-35 years | 0.00086 | 0.00005 |  |  |
| 36-45 years | 0.00093 | 0.00005 |  |  |
| 46-60 years | 0.00081 | 0.00005 |  |  |
| >60 years | 0.00015 | 0.00008 |  |  |
| Relative risk of injuries (comparator- abstainer) |  |  |  |  |
| Low risk alcohol use | 1.1 | 0.064 | Uniform | [129] |
| Hazardous alcohol use | 1.8 | 0.104 |  |  |
| Harmful alcohol use/ dependence | 2.5 | 0.144 |  |  |
| Proportion of severity of injuries |  |  |  |  |
| Mild | 0.4986 | 0.029 | Beta | [123], [130] |
| Moderate | 0.1492 | 0.009 |  |  |
| Severe | 0.0293 | 0.002 |  |  |
| Fatal | 0.3229 | 0.019 |  |  |
| Probability of mortality from mild injury |  |  |  |  |
| <20 years | 0.0109 | 0.0006 | Beta | [126] |
| 20-29 years | 0.0125 | 0.0007 |  |  |
| 30-39 years | 0.0128 | 0.0008 |  |  |
| 40-49 years | 0.0148 | 0.0009 |  |  |
| 50-59 years | 0.0225 | 0.0013 |  |  |
| 60-69 years | 0.0342 | 0.0020 |  |  |
| 70-79 years | 0.0588 | 0.0034 |  |  |
| >79 years | 0.0824 | 0.0048 |  |  |
| Probability of mortality from moderate injury |  |  |  |  |
| <20 years | 0.0370 | 0.0022 | Beta | [126] |
| 20-29 years | 0.0426 | 0.0025 |  |  |
| 30-39 years | 0.0436 | 0.0025 |  |  |
| 40-49 years | 0.0502 | 0.0029 |  |  |
| 50-59 years | 0.0746 | 0.0043 |  |  |
| 60-69 years | 0.1106 | 0.0064 |  |  |
| 70-79 years | 0.1806 | 0.0105 |  |  |
| >79 years | 0.2417 | 0.0140 |  |  |
| Probability of mortality from severe injury |  |  |  |  |
| <20 years | 0.1357 | 0.0078 | Beta | [126] |
| 20-29 years | 0.1532 | 0.0088 |  |  |
| 30-39 years | 0.1562 | 0.0090 |  |  |
| 40-49 years | 0.1758 | 0.0102 |  |  |
| 50-59 years | 0.2419 | 0.0139 |  |  |
| 60-69 years | 0.3246 | 0.0187 |  |  |
| 70-79 years | 0.4503 | 0.0259 |  |  |
| >79 years | 0.5355 | 0.0309 |  |  |
| Care seeking behaviour |  |  |  |  |
| Proportion of people with mild injury not seeking care | 0.077 | 0.0044 | Normal | [131] |
| Proportion of patients requiring long-term rehabilitation |  |  |  |  |
| Mild injury | 0.1 | 0.0058 | Normal | [125] |
| Moderate injury | 0.5 | 0.0289 |  |  |
| Severe injury | 1 | 0.0577 |  |  |
| Type of provider accessed |  |  |  |  |
| *Mild injury* |  |  |  | [121] |
| SC | 0.014 | 0.0008 | Normal |  |
| PHC | 0.020 | 0.0012 |  |  |
| CHC | 0.020 | 0.0012 |  |  |
| District Hospital | 0.132 | 0.0076 |  |  |
| Government medical college | 0.132 | 0.0076 |  |  |
| Private clinic | 0.2 | 0.0115 |  |  |
| Private hospital | 0.482 | 0.0278 |  |  |
| *Moderate and severe injury* |  |  |  |  |
| CHC | 0.027 | 0.0016 | Normal | [121] |
| District Hospital | 0.197 | 0.0114 |  |  |
| Government medical college | 0.197 | 0.0114 |  |  |
| Private Hospital | 0.578 | 0.0334 |  |  |
| Health systems treatment cost ₹ ($) |  |  |  |  |
| Primary (SC) | 184  (2.30) | 53.116 (0.675) | Gamma | [132] |
| Primary (PHC) | 358 (4.50) | 103.346 (1.314) |  |  |
| Secondary (public) | 1703 (21) | 491.533 (6.253) |  |  |
| Tertiary (public) | 30609 (389) | 8836.131 (112.418) |  |  |
| Annual OOPE for treatment (first year of accident) ₹ ($) |  |  |  |  |
| Primary (public) | 1611 (20) | 465.056 (5.916) | Gamma | [127] |
| Secondary (public) | 6526 (83) | 1883.894 (23.968) |  |  |
| Tertiary (public) | 20802 (266) | 6005.020 (76.399) |  |  |
| Private clinic | 14236 (182) | 4109.488 (52.283) |  | [133] |
| Private hospital | 93796 (1202) | 27076.573 (344.485) |  |  |
| Annual OOPE (subsequent years of accident) ₹ ($) |  |  |  |  |
| Public | 430 (5.50) | 124.130 (1.579) | Gamma | [127] |
| Private | 2869 (37) | 828.209 (10.537) |  |  |
| Utility values (Males) |  |  |  |  |
| *First month of injury* |  |  |  |  |
| Mild injury | 0.862 | 0.0995 | Beta | [128] |
| Moderate injury | 0.746 | 0.0861 |  |  |
| Severe injury | 0.597 | 0.0689 |  |  |
| *Second and third month of injury* |  |  |  |  |
| Mild injury | 0.952 | 0.1099 | Beta |  |
| Moderate injury | 0.894 | 0.1032 |  |  |
| Severe injury | 0.813 | 0.0939 |  |  |
| *Fourth to 11th month of injury* |  |  |  |  |
| Mild injury | 0.983 | 0.1135 | Beta |  |
| Moderate injury | 0.973 | 0.1124 |  |  |
| Severe injury | 0.935 | 0.1080 |  |  |
| *12 months of injury and later* |  |  |  |  |
| Mild injury | 0.995 | 0.1149 | Beta |  |
| Moderate injury | 0.987 | 0.1140 |  |  |
| Severe injury | 0.959 | 0.1107 |  |  |
| Utility values (females) |  |  |  |  |
| *First month of injury* |  |  |  |  |
| Mild injury | 0.862 | 0.0995 | Beta |  |
| Moderate injury | 0.759 | 0.0876 |  |  |
| Severe injury | 0.584 | 0.0674 |  |  |
| *Second and third month of injury* |  |  |  |  |
| Mild injury | 0.957 | 0.1105 | Beta |  |
| Moderate injury | 0.89 | 0.1028 |  |  |
| Severe injury | 0.791 | 0.0913 |  |  |
| *Fourth to 11th month of injury* |  |  |  |  |
| Mild injury | 0.982 | 0.1134 | Beta |  |
| Moderate injury | 0.978 | 0.1129 |  |  |
| *Severe injury* | 0.934 | 0.1078 |  |  |
| 12 months of injury and later |  |  |  |  |
| Mild injury | 0.987 | 0.1140 | Beta |  |
| Moderate injury | 0.979 | 0.1130 |  |  |
| Severe injury | 0.982 | 0.1134 |  |  |

^RTA: Road traffic accident injuries, AUD: Alcohol Use Disorder, SC: Sub-centre, PHC: Primary Health Centre, CHC: Community Health Centre, ₹: Indian National Rupee, OOPE: Out-of-pocket expenditure^

### *Alcohol related liver disease (ALD): Model structure and parameters*

It was assumed that 85% of the individuals with harmful alcohol use, and alcohol dependence would develop steatosis, as suggested by the clinical experts. The model structure for ALD was adapted from Delacote et al [134] (Figure S3). The health states considered in the model included steatosis, alcoholic steatohepatitis (ASH) with or without fibrosis, compensated cirrhosis, decompensated cirrhosis with ascites, variceal bleeding, hepatic encephalopathy, hepatocellular carcinoma, and mortality. It was assumed that individuals could transition from a state of steatosis to a healthy state, contingent upon alcohol abstinence, as supported by existing literature. Moreover, while transitions to all-cause mortality were assumed to be possible from all health states, liver-related mortality was specifically considered only from cirrhosis and hepatocellular carcinoma.

Figure S4. Markov state transition model for Alcohol Related Liver Disease

The care-seeking patterns for ALD were obtained from the National Sample Survey of India [121] (Table S4). Treatment protocols for each health state were simulated in accordance with the standard clinical guidelines. Treatment costs were determined using data from national sources (Table S4), with separate calculations for different levels of care and distinctions between the public and private sectors.

Table S5. Base value of parameters for markov model on Alcohol Related Liver Disease and data sources

| Parameter | Base value | Standard error | Distribution | Source |
| --- | --- | --- | --- | --- |
| Transition probabilities |  |  |  |  |
| Normal to steatosis | 0.85 | 0.0491 | Beta | [134], [135] |
| Steatosis to normal | 0.26 | 0.0150 |  |  |
| Steatosis to fibrosis | 0.02 | 0.0012 |  |  |
| Steatosis with fibrosis to steatosis | 0.26 | 0.0150 |  |  |
| Steatosis to alcoholic steatohepatitis (ASH) | 0.02 | 0.0012 |  |  |
| ASH to ASH with fibrosis | 0.14 | 0.0081 |  |  |
| Steatosis with fibrosis to compensated cirrhosis | 0.08 | 0.0046 |  |  |
| Steatosis to compensated cirrhosis | 0.08 | 0.0046 |  |  |
| ASH to compensated cirrhosis | 0.1 | 0.0058 |  |  |
| Compensated cirrhosis to decompensated cirrhosis with ascites | 0.0837 | 0.0048 |  |  |
| Compensated cirrhosis to decompensated cirrhosis with variceal bleeding | 0.0465 | 0.0027 |  |  |
| Compensated cirrhosis to decompensated cirrhosis with hepatic encephalopathy | 0.11 | 0.0064 |  |  |
| Decompensated cirrhosis with ascites to ascites and variceal bleeding | 0.0657 | 0.0038 |  |  |
| Decompensated cirrhosis with variceal bleeding to ascites and variceal bleeding | 0.099 | 0.0057 |  |  |
| Decompensated cirrhosis with ascites to hepatic encephalopathy | 0.0597 | 0.0034 |  |  |
| Decompensated cirrhosis with ascites to mortality | 0.151 | 0.0087 |  |  |
| Decompensated cirrhosis with variceal bleeding to hepatic encephalopathy | 0.0735 | 0.0042 |  |  |
| Decompensated cirrhosis with variceal bleeding to mortality | 0.0811 | 0.0047 |  |  |
| Decompensated cirrhosis with variceal bleeding and ascites to hepatic encephalopathy | 0.175 | 0.0101 |  |  |
| Decompensated cirrhosis with variceal bleeding and ascites to mortality | 0.175 | 0.0101 |  |  |
| Hepatic encephalopathy to mortality | 0.189 | 0.0109 |  |  |
| Hepatocellular carcinoma to mortality | 0.32 | 0.0185 |  |  |
| Care seeking behaviour- Steatosis, ASH, compensated cirrhosis |  |  |  |  |
| District hospital | 0.1285 | 0.0074 | Normal | [121] |
| Government medical college | 0.1285 | 0.0074 |  |  |
| Private clinic | 0.357 | 0.0206 |  |  |
| Private hospital | 0.386 | 0.0223 |  |  |
| Care seeking behaviour- Decompensated cirrhosis, Hepatocellular carcinoma |  |  |  |  |
| Government medical college | 0.6 | 0.0346 | Normal | [121] |
| Private hospital | 0.4 | 0.0231 |  |  |
| Per Diagnostic Cost ₹ ($) |  |  |  |  |
| Complete hemogram | 138 (1.75) | 39.837 (0.506) | Gamma | [115] |
| Liver Function Test | 275 (3.50) | 79.386 (1.010) |  |  |
| Renal Function Test | 261 (3.32) | 75.344 (0.958) |  |  |
| Ultrasound | 300 (3.81) | 86.603 (1.101) |  |  |
| Prothrombin time | 119 (1.51) | 34.352 (0.437) |  |  |
| Fibro scan | 1150 (14.63) | 331.976 (4.223) |  |  |
| Alpha-fetoprotein | 345 (4.38) | 99.593 (1.267) |  |  |
| Ascitic fluid analysis | 1800 (23) | 519.615 (6.610) |  |  |
| Endoscopy | 15000 (191) | 4330.127 (55.090) |  |  |
| PET scan | 21000 (267) | 6062.178  (77.126) |  |  |
| Annual treatment costs ₹ ($) |  |  |  |  |
| Baclofen | 257 (3.26) | 74.190 (0.943) | Gamma | [114] |
| Carvedilol | 358 (4.55) | 103.346 (1.314) |  |  |
| Propranolol | 516 (6.56) | 148.956 (1.895) |  |  |
| Lactulose | 378 (4.80) | 109.119 (1.380) |  |  |
| Rifaximin | 8006 (102) | 2311.133 (29.403) |  |  |
| Furosemide and spironolactone | 2127 (27) | 614.012 (7.811) |  |  |
| Albumin | 1806 (23) | 521.347 (6.632) |  |  |
| Branch chain amino acids | 1203 (15) | 347.276 (4.418) |  |  |
| Ablation therapy | 75000 (954) | 21650.640 (275.453) |  | [136] |
| Trans arterial Chemoembolization | 100000  (1272) | 28867.510 (367.271) |  |  |
| Surgery | 25000  (318) | 7216.878 (91.817) |  |  |
| Palliative care | 36000  (458) | 10392.3 (132.217) |  |  |
| Per bed day hospitalization cost (District Hospital) | 1439  (18) | 415.404 (5.285) |  | [114] |
| Per bed day hospitalization cost (Medical College) | 1951  (25) | 563.205 (7.165) |  |  |
| Per bed day hospitalization cost in ICU setting | 11068  (140) | 3195.056 (40.655) |  | [137] |
| Per bed day hospitalization cost in HDU setting | 12697  (161) | 3665.308 (46.632) |  |  |
| OOPE per OPD visit (ALD excluding HCC) ₹ ($) |  |  |  |  |
| Public facility | 344 (4.37) | 99.304 (1.263) | Gamma | [121] |
| Private clinic (OPD) | 92 (1.17) | 26.558 (0.337) |  |  |
| Private hospital (OPD) | 315 (4) | 90.933 (1.156) |  |  |
| OOPE per hospitalization (ALD excluding HCC) ₹ ($) |  |  |  |  |
| Public facility | 2780 (35) | 802.517 (10.210) | Gamma | [121] |
| Private hospital | 36130 (459) | 10429.83 (132.695) |  |  |
| OOPE per hospitalization (HCC) ₹ ($) |  |  |  |  |
| Public facility (OPD) | 388 (5) | 112.006 (1.425) | Gamma | [138] |
| Private clinic (OPD) | 582 (7) | 168.009 (2.137) |  |  |
| Public facility (IPD) | 5941 (75) | 1715.019 (21.819) |  |  |
| Utility values |  |  |  |  |
| Steatosis/ steatosis with fibrosis | 0.98 | 0.113 | Beta | [139] |
| ASH/ ASH with fibrosis | 0.76 | 0.088 |  |  |
| Compensated cirrhosis | 0.7 | 0.081 |  |  |
| Decompensated cirrhosis | 0.6 | 0.069 |  |  |
| Hepatocellular carcinoma | 0.6 | 0.069 |  | [138] |

^ASH: Alcoholic steatohepatitis, PET: Positron Emission Tomography, ICU: Intensive Care Unit, HDU: High Dependency Unit, OPD: Outpatient Department, HCC: Hepatocellular Carcinoma, OOPE: Out-of-pocket expenditure, ALD: Alcohol-related Liver Disease, ₹: Indian National Rupee^

### Head and Neck Cancers: Model structure and parameters

The model structure for simulation of natural history of progression of head and neck cancers (HNC) was developed based on published literature [140, 141] (Figure S4). Age-specific incidence rates of HNC were obtained from the Indian study which synthesized data from 37 population-based cancer registries across the country [142]. An additional risk of development of HNC was applied in alcohol consumers, based on the reported odds of acquiring HNCs in Indian men with different levels of alcohol consumption (Figure S4) [143].

It was assumed that AUD screening would lead to detection of HNCs in early stages, at a similar rate as oral cancer screening. Hence, the proportion of patients in different stages of cancer in usual care and screening scenario were derived from a cluster randomized trial on effectiveness of oral cancer screening in India (Table S5) [144]. The annual risk of cancer associated deaths in different stages of cancer was informed by a recent hospital-based study on 1,895 patients with oral cancer [145].

To derive the utility values for each Markov health state, we relied on data from a comprehensive cross-sectional study conducted across multiple cancer centres, which provide services to cancer patients from 17 states of India [138]. This dataset included HRQoL information from 687,198 patients diagnosed with cancers, measured using the EQ-5D-5L instrument. Further, the care seeking patterns of the cancer patients were simulated, based on the data of the most recent round of the National Sample Survey [121].

For the estimation of medical costs, we used the provider payment rates outlined in the 2022 Health Benefit Package of the government-funded health insurance scheme [136]. These rates cover all aspects of care, including surgical, medical, and radiation treatment, as well as pre- and post-hospitalization services such as consultations, diagnostic tests, medications, and follow-up care within 15 days of hospital discharge. Data on non-medical expenditures were sourced from the National Cancer Database of Costs and Quality of Life in India.

Figure S5. Markov state transition model for Head and Neck Cancer

Table S6. Base value of parameters for markov model on Head and Neck Cancer and data sources

| Parameter | Base Value | Standard error | Distribution | Source |
| --- | --- | --- | --- | --- |
| Incidence of HNC- males |  |  |  |  |
| 0-19 years | 0.000002 | 0.0000001 | Beta | [142] |
| 20-39 years | 0.000077 | 0.000004 |  |  |
| 40-59 years | 0.000526 | 0.00003 |  |  |
| 60 years and above | 0.001149 | 0.00006 |  |  |
| Incidence of HNC- females |  |  |  |  |
| 0-19 years | 0.000001 | 0.00000005 | Beta | [142] |
| 20-39 years | 0.00002 | 0.000001 |  |  |
| 40-59 years | 0.000158 | 0.000009 |  |  |
| 60 years and above | 0.000369 | 0.00002 |  |  |
| Proportion of males (usual care) |  |  |  |  |
| Stage I | 0.1596 | 0.0092 | Normal | [144] |
| Stage II | 0.1357 | 0.0078 |  |  |
| Stage III | 0.2245 | 0.0129 |  |  |
| Stage IV | 0.4803 | 0.0277 |  |  |
| Proportion of females (usual care) |  |  |  |  |
| Stage I | 0.1441 | 0.0083 | Normal | [144] |
| Stage II | 0.1225 | 0.0071 |  |  |
| Stage III | 0.2330 | 0.0134 |  |  |
| Stage IV | 0.5005 | 0.0289 |  |  |
| Proportion of males (screening scenario) |  |  |  |  |
| Stage I | 0.3058 | 0.0177 | Normal | [144] |
| Stage II | 0.2038 | 0.0118 |  |  |
| Stage III | 0.1686 | 0.0097 |  |  |
| Stage IV | 0.3555 | 0.0205 |  |  |
| Proportion of females (screening scenario) |  |  |  |  |
| Stage I | 0.2761 | 0.0159 | Normal | [144] |
| Stage II | 0.1841 | 0.0106 |  |  |
| Stage III | 0.1750 | 0.0101 |  |  |
| Stage IV | 0.3705 | 0.0214 |  |  |
| Annual probability of cancer death |  |  |  |  |
| Stage I | 0.02947 | 0.0017 | Beta | [145] |
| Stage II | 0.06095 | 0.0035 |  |  |
| Stage III | 0.08601 | 0.0050 |  |  |
| Stage IV | 0.13272 | 0.0077 |  |  |
| Loco-regional recurrence | 0.613 | 0.0354 |  | [140] |
| Odds of developing HNC in population with alcohol consumption |  |  |  |  |
| Low risk use | 1.23 | 0.0710 | Uniform | [143] |
| Hazardous use | 2.4 | 0.1386 |  |  |
| Harmful use | 2.98 | 0.1721 |  |  |
| Alcohol dependence | 2.98 | 0.1721 |  |  |
| Progression probabilities |  |  |  |  |
| Stage I/II to Loco-regional recurrence | 0.043 | 0.0025 | Beta | [140, 146-147] |
| Stage III/IV to Loco-regional recurrence | 0.065 | 0.0038 |  |  |
| Stage I/II to Distant metastasis | 0.004 | 0.0003 |  |  |
| Stage III/IV to Distant metastasis | 0.0136 | 0.0008 |  |  |
| Loco-regional recurrence to Distant metastasis | 0.076 | 0.0044 |  |  |
| Loco-regional recurrence to Salvage (neck dissection) | 0.083 | 0.0048 |  |  |
| Loco-regional recurrence to Salvage (No neck dissection) | 0.146 | 0.0084 |  |  |
| Salvage surgery to Loco-regional recurrence (neck dissection) | 0.136 | 0.0079 |  |  |
| Salvage surgery to Loco-regional recurrence (No neck dissection) | 0.115 | 0.0067 |  |  |
| Salvage surgery to Distant metastasis (neck dissection) | 0.032 | 0.0018 |  |  |
| Salvage surgery to Distant metastasis (No neck dissection) | 0.030 | 0.0017 |  |  |
| Utility values |  |  |  |  |
| Stage I | 0.738 | 0.0852 | Beta | [138] |
| Stage II | 0.704 | 0.0813 |  |  |
| Stage III | 0.684 | 0.0789 |  |  |
| Stage IV | 0.48 | 0.0554 |  |  |
| Loco-regional recurrence | 0.659 | 0.0761 |  |  |
| Distant metastasis | 0.508 | 0.0587 |  |  |
| Salvage surgery | 0.264 | 0.0305 |  |  |
| Care seeking behaviour |  |  |  |  |
| Public | 0.4 | 0.0231 | Normal | [121] |
| Private | 0.6 | 0.0346 |  |  |
| Health systems cost ₹ ($) |  |  |  |  |
| Cisplatin 40mg/m^2^ | 2900 (37) | 837.158 (10.650) | Gamma | [136] |
| Cisplatin 100mg/m^2^ | 11950 (153) | 3449.668 (43.888) |  |  |
| 2D External Beam Radiotherapy | 13390 (171) | 3865.360 (49.177) |  |  |
| 3D-CRT | 25540 (327) | 7372.763 (93.801) |  |  |
| IMRT | 85050 (1090) | 24551.820 (312.364) |  |  |
| High end radiological diagnostic | 5560 (71) | 1605.034 (20.420) |  |  |
| Paclitaxel 175mg/m^2^ | 14380 (184) | 4151.148 (52.813) |  |  |
| Paclitaxel + Carboplatin 175mg/m^2^ | 17540 (225) | 5063.362  (64.419) |  |  |
| Basic Supportive Care | 10215 (131) | 2948.817  (37.516) |  |  |
| Modified Neck Dissection | 36870 (472) | 10643.452  (135.931) |  |  |
| Selective Neck Dissection | 26040 (333) | 7517.101  (95.637) |  |  |
| Salvage surgery | 60550 (776) | 17479.279  (222.382) |  |  |
| Non-medical expenditure ₹ ($) |  |  |  |  |
| Per bed day hospitalization | 5941 (76) | 1715.019  (21.819) | Gamma | [138] |
| Per OPD visit in public facility | 388 (5) | 112.006  (1.425) |  |  |
| Per OPD visit in private facility | 582 (7.40) | 168.009 (2.137) |  |  |

^HNC: Head and Neck Cancer, CRT: Chemoradiotherapy, IMRT: Intensity-Modulated Radiation Therapy, OPD: Outpatient Department, ₹: Indian National Rupee^

# **Section 5. Results**

Table S7. Undiscounted life years, and QALYs in usual care and screening scenario

|  | **Life years (billions)** | | **QALYs**  **(billions)** | **Life-year gain**  **(millions)** | **QALY gain**  **(millions)** |
| --- | --- | --- | --- | --- | --- |
| **Usual care** | | 5.699  (5.693- 5.706) | 5.626  (5.618- 5.634) |  |  |
| **Screening programme** | | 5.826  (5.819- 5.833) | 5.768  (5.761- 5.776) | 126.31  (126.05- 126.68) | 142.37 (142.355- 142.376) |

Table S8. Incident cases of alcohol attributable conditions at population level (%)

|  | % cases at population level | | Absolute reduction (%) |
| --- | --- | --- | --- |
|  | Usual Care | Screening programme |  |
| Road traffic accidents | 0.26 | 0.18 | 0.08 |
| Head and neck cancer | 0.03 | 0.02 | 0.01 |
| Alcohol related liver disease | 11.8 | 9.16 | 2.68 |
| Total | 12.13 | 9.36 | 2.77 |

Table S9. State-wise reduction in alcohol attributable conditions

| States | Alcohol attributable conditions per 100,000 population | | Reduction in alcohol attributable conditions per 100,000 population | %age reduction |
| --- | --- | --- | --- | --- |
|  | Usual care | Screening |  |  |
| Bihar | 488 | 330 | 158 | 32.44% |
| Rajasthan | 1,645 | 1,405 | 240 | 14.57% |
| Gujarat | 2,898 | 2,233 | 665 | 22.95% |
| Jammu and Kashmir | 2,921 | 2,191 | 730 | 24.98% |
| Maharashtra | 4,439 | 3,701 | 738 | 16.63% |
| Karnataka | 5,554 | 4,768 | 786 | 15.55% |
| Telangana | 7,463 | 6,629 | 834 | 12.53% |
| Andhra Pradesh | 12,830 | 11,698 | 1,132 | 10.03% |
| Jharkhand | 3,601 | 2,381 | 1,219 | 33.87% |
| Assam | 6,944 | 5,407 | 1,536 | 22.12% |
| Himachal Pradesh | 6,947 | 4,672 | 2,275 | 32.75% |
| Kerala | 8,052 | 5,306 | 2,746 | 34.10% |
| West Bengal | 9,669 | 6,729 | 2,940 | 30.40% |
| Odisha | 12,921 | 9,765 | 3,155 | 24.42% |
| Tamil Nadu | 25,581 | 22,381 | 3,200 | 12.51% |
| Madhya Pradesh | 13,667 | 9,832 | 3,836 | 28.06% |
| Uttar Pradesh | 20,051 | 15,986 | 4,064 | 21.93% |
| Uttarakhand | 14,917 | 10,396 | 4,521 | 30.31% |
| Delhi | 18,307 | 13,483 | 4,823 | 26.35% |
| Haryana | 16,378 | 11,316 | 5,063 | 30.91% |
| Punjab | 24,025 | 17,544 | 6,481 | 26.98% |
| Chhattisgarh | 27,447 | 20,384 | 7,063 | 25.73% |

Table S10. Undiscounted Direct costs (in ₹ billions)

|  | Usual care (₹) | Usual care ($) | Screening programme (₹) | Screening programme ($) |
| --- | --- | --- | --- | --- |
| Screening and treatment | 1,472  (1,458- 1,487) | 18.72  (18.54-18.91) | 8,670  (8,592- 8,749) | 110.30  (109.31-111.31) |
| Road traffic accident injuries | 216  (213- 219) | 2.74  (2.70-2.78) | 147  (145- 149) | 1.87  (1.84-1.89) |
| Head and Neck cancers | 214  (212- 216) | 2.72  (2.69-2.74) | 141  (139- 142) | 1.79  (1.76-1.80) |
| Alcohol Related Liver Disease | 18,556  (18389- 18,724) | 236  (234-238) | 14,555  (14,415- 14,695) | 185  (183-187) |
| Total Direct Costs | **20,459**  **(20,291- 20,627)** | **260**  **(258-262)** | **23,513**  **(23,359- 23,668)** | **299**  **(297-301)** |

Table S11. Discounted direct costs with and without AUD screening (in $ billions)

|  | Usual care* | Screening programme | Difference between usual care and screening programme |
| --- | --- | --- | --- |
| Screening and treatment of AUD | 19  (18-20) | 111  (110- 112) | 92  (91- 93) |
| Treatment of Road traffic accident injuries | 2.60  (2.50- 2.70) | 1.80  (1.70- 1.90) | -0.83  (0.82- 0.84) |
| Treatment of Head and Neck cancers | 2.60  (2.50- 2.70) | 1.70  (1.60- 180) | -0.92  (0.91- 0.93) |
| Treatment of Alcohol Related Liver Disease | 217  (216- 218) | 170  (169- 171) | -46  (45- 44) |
| Total Direct Costs | 241  (240- 242) | 285  (284-286) | 43  (42- 44) |

^*No screening costs AUD: Alcohol use disorder^

Table S12. Undiscounted Indirect costs (in ₹ and $ billions)

|  | Usual care (₹) | Usual care  ($) | Screening programme (₹) | | Screening programme ($) |
| --- | --- | --- | --- | --- | --- |
| Road traffic accident injuries | 5,363  (5,333- 5,393) | 68  (67-68) | | 3,666  (3,643- 3,689) | 46  (45-47) |
| Head and Neck cancers | 2,849  (2,833- 2,864) | 36.24  (36.04-36.43) | | 1,848  (1,837- 1,859) | 23  (22-24) |
| Alcohol Related Liver Disease | 22,242  (22,144- 22,340) | 282  (281-283) | | 17,495  (17,403- 17,587) | 222  (221-223) |
| Total Indirect Costs | **30,454**  **(30,349- 30,559)** | **387**  **(386-388)** | | **23,009**  **(22,905- 23,114)** | **292**  **(291-293)** |

Table S13. Indirect costs (billions $) associated with premature mortality and loss of productivity

|  | **Indirect Costs** | | | **Total** |
| --- | --- | --- | --- | --- |
|  | RTA | HNC | ALD |  |
| **Usual care** | 68  (67- 69) | 33  (32- 34) | 257  (256- 258) | 360  (359- 361) |
| **Screening** | 47  (46- 48) | 21  (20- 22) | 202  (201- 202) | 271  (270- 272) |
| **Indirect Costs averted** | 21  (20- 22) | 12  (11- 13) | 55  (54- 56) | 88  (87- 89) |

^RTA: Road traffic accident injuries, HNC: Head and Neck Cancer, ALD: Alcohol related liver disease.^

Table S14. Discounted life years, QALYs, costs and ICURs, using NFHS-V prevalence data

| **Strategies** | **Life Years*** | **QALYs*** | **Direct Cost***  **₹ ($)** | **Indirect cost* ₹ ($)** | **ICUR: ₹ ($)**  **(Abridged societal perspective)** | **ICUR: ₹ ($)**  **(Societal perspective)** |
| --- | --- | --- | --- | --- | --- | --- |
| **Usual care** | 3.42 | 3.38 | 15,444  (196) | 22,300  (283) |  |  |
| **Screening** | 3.46 | 3.42 | 20,666  (262) | 16,672  (212) | 100,608  (1280) | -21,900  (278) |

*In billions. Bracket values in costs and ICURs present values on US$

Table S15. Discounted life years, QALYs, costs and ICURs, if screening is conducted in 30-74 years age group

|  | **Life Years*** | **QALYs*** | **Direct Cost***  **₹ ($)** | **Indirect cost***  **₹ ($)** | **ICUR: ₹ ($)**  **(Abridged societal perspective)** | **ICUR: ₹ ($)**  **(Societal perspective)** |
| --- | --- | --- | --- | --- | --- | --- |
| Usual care | 4.121 | 3.991 | 26,955  (342) | 24,910  (316) |  |  |
| Annual Screening in 30-74 years | 4.128 | 4.025 | 27,936  (355) | 19,325  (245) | **50,811**  (646) | **-2,75,538**  (3505) |

*In billions

Table S16. Impact of varying rates of treatment and treatment compliance on ICUR values (₹)*

| Treatment Rate in screening scenario | Proportion with treatment compliance after 3 months of treatment | | | |
| --- | --- | --- | --- | --- |
|  | 66% | 46% | 26% | 14% |
| 85% | 55,588 | 87,673 | 148,528 | 233,037 |
| 65% | 70,697 | 104,967 | 178,203 | 283,279 |
| 45% | 94,727 | 139,676 | 239,451 | 391,856 |
| 25% | 164,351 | 244,172 | 441,440 | 809,663 |
| 15% | 292,376 | 454,108 | 954,182 | 2,603,840 |

*Based on abridged societal perspective

Green boxes represent that screening in 15-74 years population remains cost-effective at the specific treatment rate and treatment compliance. Orange boxes depict that the screening intervention is cost-ineffective at threshold of per capita GDP.

Table S17. Comparison of life years of people with alcohol use without co-morbidity from the model with SRS life tables

| **Age group (years)** | **Life years (Model)** | **Life years (SRS life tables)** |
| --- | --- | --- |
| 15-19 | 56.9 |  |
| 20-24 | 52.4 | 53.4 |
| 25-29 | 47.8 |  |
| 30-34 | 43.2 | 44.0 |
| 35-39 | 39.0 |  |
| 40-44 | 35.7 | 34.8 |
| 45-49 | 30.5 |  |
| 50-54 | 26.5 | 26.0 |
| 55-59 | 22.9 |  |
| 60-64 | 19.9 | 18.3 |
| 65-69 | 17.0 |  |
| 70-74 | 12.7 | 11.9 |

Table S18. Comparison of discounted life year and QALY gain attributable to screening interventions for alcohol use disorders

| Author (Country, Time horizon, Discounting) | Life year gain per person | QALY gain per person | Conclusion |
| --- | --- | --- | --- |
| Angus et al (Italy, 30 years, 3.5%) [35] |  | 0.019 | Cost-effective |
| Zur and Zariac (Canada, Lifetime, 3%) [48] | 0.046 | 0.057 | Cost-effective |
| Purshouse et al (England, 1 year, NA) [45] |  | 0.004 | Cost-effective |
| Barbosa et al (US, 1 year, NA) [50] |  | 0.008 | Cost-saving |
| Current study (India, Lifetime, 3%) | 0.048 | 0.059 | Cost-effective |

Grey boxes indicate non-reporting of information in the study. US: United States of America

Table S19. Compliance to CHEERS Checklist [148]

|  | **Item** | **Guidance for Reporting** | **Reported in section** |
| --- | --- | --- | --- |
| **TITLE** | | |  |
| Title | 1 | Identify the study as an economic evaluation and specify the interventions being compared. | Page 1 |
| **ABSTRACT** | | |  |
| Abstract | 2 | Provide a structured summary that highlights context, key methods, results and alternative analyses. | Page 2, 3 |
| **INTRODUCTION** | | |  |
| Background and objectives | 3 | Give the context for the study, the study question and its practical relevance for decision making in policy or practice. | Page 3, 4 |
| **METHODS** | | |  |
| Health economic  analysis plan | 4 | Indicate whether a health economic analysis plan was developed and where available. |  |
| Study population | 5 | Describe characteristics of the study population (such as age range, demographics, socioeconomic, or clinical characteristics). | Page 6 |
| Setting and location | 6 | Provide relevant contextual information that may influence findings. | Page 5,7 |
| Comparators | 7 | Describe the interventions or strategies being compared and why chosen. | Page 5 (manuscript), Page 18 of the supplement |
| Perspective | 8 | State the perspective(s) adopted by the study and why chosen. | Page 5 |
| Time horizon | 9 | State the time horizon for the study and why appropriate. | Page 5 |
| Discount rate | 10 | Report the discount rate(s) and reason chosen. | Page 6 |
| Selection of outcomes | 11 | Describe what outcomes were used as the measure(s) of benefit(s) and harm(s). | Page 8 |
| Measurement of outcomes | 12 | Describe how outcomes used to capture benefit(s) and harm(s) were measured. | Page 6-8 |
| Valuation of outcomes | 13 | Describe the population and methods used to measure and value outcomes. | Page 5-8 |
| Measurement and valuation of resources  and costs | 14 | Describe how costs were valued. | Page 8 |
| Currency, price date, and conversion | 15 | Report the dates of the estimated resource quantities and unit costs, plus the currency and year of conversion. | Page 8 |
| Rationale and  description of model | 16 | If modelling is used, describe in detail and why used. Report if the model is publicly available and where it can be accessed. | Page 5 |
| Analytics and assumptions | 17 | Describe any methods for analysing or statistically transforming data, any extrapolation methods, and approaches for validating any model used. | Page 5-9 |
| Characterizing heterogeneity | 18 | Describe any methods used for estimating how the results of the study vary for sub-groups. | Page 9 |
| Characterizing  distributional effects | 19 | Describe how impacts are distributed across different individuals or adjustments made to reflect priority populations. |  |
| Characterizing uncertainty | 20 | Describe methods to characterize any sources of uncertainty in the analysis. | Page 9 |
| Approach to engagement with patients and others affected by the study | 21 | Describe any approaches to engage patients or service recipients, the general public, communities, or stakeholders (e.g., clinicians or payers) in the design of the study. |  |
| **RESULTS** | | |  |
| Study parameters | 22 | Report all analytic inputs (e.g., values, ranges, references) including uncertainty or distributional assumptions. | Page 5-9 (main manuscript), 21-40 (supplement) |
| Summary of main results | 23 | Report the mean values for the main categories of costs and outcomes of interest and summarise them in the most appropriate overall measure. | Page 9- 11 (main manuscript), 40-43 (supplement) |
| Effect of uncertainty | 24 | Describe how uncertainty about analytic judgments, inputs, or projections affect findings. Report the effect of choice of discount rate and time horizon, if applicable. | Page 11(main manuscript), 43-44 (supplement) |
| Effect of engagement with patients and others affected by the study | 25 | Report on any difference patient/service recipient, general public, community, or stakeholder involvement made to the approach or findings of the study |  |
| **DISCUSSION** | | |  |
| Study findings, limitations, generalizability, and current knowledge | 26 | Report key findings, limitations, ethical or equity considerations not captured, and how these could impact patients, policy, or practice. | Page 11- 15 |
| **OTHER RELEVANT INFORMATION** | | | |
| Source of funding | 27 | Describe how the study was funded and any role of the funder in the identification, design, conduct, and reporting of the analysis | Page 1 |
| Conflicts of interest | 28 | Report authors conflicts of interest according to journal or  International Committee of Medical Journal Editors requirements. | Page 1 |

# **Section 6. References**

1. Barbosa C, Cowell A, Dowd W, Landwehr J, Aldridge A, Bray J. The cost-effectiveness of brief intervention versus brief treatment of screening, brief intervention and referral to treatment (SBIRT) in the United States. Addiction 2017; 112 (suppl 2): 73–81
2. Barrett B, Byford S, Crawford MJ, Patton R, Drummond C, Henry JA, Touquet R. Cost-effectiveness of screening and referral to an alcohol health worker in alcohol misusing patients attending an accident and emergency department: a decision-making approach. Drug Alcohol Depend. 2006 Jan 4;81(1):47-54.
3. Hunter R, Wallace P, Struzzo P, Vedova RD, Scafuri F, Tersar C, et al.Randomised controlled non-inferiority trial of primary care-based facilitated access to an alcohol reduction website: cost-effectiveness analysis. BMJOpen 2017; 7(11): e014577.
4. Kouimtsidis C, Bosco A, Scior K, Baio G, Hunter R, Pezzoni V, Mcnamara E, Hassiotis A. A feasibility randomised controlled trial of extended brief intervention for alcohol misuse in adults with mild to moderate intellectual disabilities living in the community; The EBI-LD study. Trials. 2017 May 12;18(1):216.
5. Li T, Waters TM, Kaplan EK, Kaplan CM, Nyarko KA, Derefinko KJ, et al. Economic analyses of an alcohol misconduct prevention program in a military setting. Mil Med 2017; 182(1): e1562–e7.
6. Mundt MP, French MT, Roebuck MC, Manwell LB, Barry KL. Brief physician advice for problem drinking among older adults: an economic analysis of costs and benefits. J Stud Alcohol 2005; 66(3): 389–94
7. Shakeshaft AP, Bowman JA, Burrows S, Doran CM, Sanson-Fisher RW. Community-based alcohol counselling: a randomized clinical trial. Addiction. 2002 Nov;97(11):1449-63.
8. Watson H, Godfrey C, McFadyen A, McArthur K, Stevenson M, Holloway A. Screening and brief intervention delivery in the workplace to reduce alcohol related harm: a pilot randomized controlled trial. Int J Nurs Stud 2015; 52(1): 39–48.
9. Wutzke SE, Shiell A, Gomel MK, Conigrave KM. Cost effectiveness of brief interventions for reducing alcohol consumption. Soc Sci Med. 2001 Mar;52(6):863-70.
10. Humphreys K, Moos RH. Reduced substance-abuse-related health care costs among voluntary participants in Alcoholics Anonymous. Psychiatr Serv. 1996 Jul;47(7):709-13.
11. Agus A, McKay M, Cole J, Doherty P, Foxcroft D, Harvey S, et al. Cost-effectiveness of a combined classroom curriculum and parental intervention: economic evaluation of data from the steps towards alcohol misuse prevention programme cluster randomised controlled trial. BMJ Open 2019; 9(7): e027951.
12. Drost RM, Paulus AT, Jander AF, et al. A web-based computer-tailored alcohol prevention program for adolescents: cost-effectiveness and intersectoral costs and benefits. J Med Internet Res 2016; 18(4): e93.
13. Fleming MF, Mundt MP, French MT, Manwell LB, Stauffacher EA, Barry KL. Brief physician advice for problem drinkers: long-term efficacy and benefit-cost analysis. Alcoholism 2002; 26(1): 36–43
14. Lindholm L. Alcohol advice in primary health care--is it a wise use of resources? Health Policy. 1998 Jul;45(1):47-56.
15. Nadkarni A, Weiss HA, Weobong B, McDaid D, Singla DR, Park AL, et al. Sustained effectiveness and cost-effectiveness of counselling for alcohol problems, a brief psychological treatment for harmful drinking in men, delivered by lay counsellors in primary care: 12-month follow-up of a randomised controlled trial. PLoS Med 2017; 14(9): e1002386
16. Goetzel RZ, Tabrizi M, Henke RM, Benevent R, Brockbank CvS, Stinson K, et al. Estimating the return on investment from a health risk management program offered to small Colorado-based employers. J Occup Environ Med 2014; 56 (5): 554.
17. Sobell LC, Sobell MB, Leo GI, Agrawal S, Johnson-Young L, Cunningham JA. Promoting self-change with alcohol abusers: a community-level mail intervention based on natural recovery studies. Alcohol Clin Exp Res. 2002 Jun;26(6):936-48.
18. Månsdotter AM, Rydberg MK, Wallin E, Lindholm LA, Andréasson S. A costeffectiveness analysis of alcohol prevention targeting licensed premises. Eur J Public Health 2007; 17(6): 618–23
19. Rehm J, Patra J, Gnam WH, Sarnocinska-Hart A, Popova S. Avoidable cost of alcohol abuse in Canada. Eur Addict Res 2011; 17(2): 72–9
20. Cobiac L, Vos T, Doran C, Wallace A. Cost-effectiveness of interventions to prevent alcohol-related disease and injury in Australia. Addiction. 2009 Oct;104(10):1646-55.
21. Chisholm D, Moro D, Bertram M, Pretorius C, Gmel G, Shield K, Rehm J. Are the "Best Buys" for Alcohol Control Still Valid? An Update on the Comparative Cost-Effectiveness of Alcohol Control Strategies at the Global Level. J Stud Alcohol Drugs. 2018 Jul;79(4):514-522. PMID: 30079865.
22. Brennan A, Meng Y, Holmes J, Hill-McManus D, Meier PS. Potential benefits of minimum unit pricing for alcohol versus a ban on below cost selling in England 2014: modelling study. BMJ 2014; 349: g5452.
23. Chisholm D, Rehm J, Van Ommeren M, Monteiro M. Reducing the global burden of hazardous alcohol use: a comparative cost-effectiveness analysis. J Stud Alcohol 2004; 65(6): 782–93
24. Purshouse RC, Meier PS, Brennan A, Taylor KB, Rafia R. Estimated effect of alcohol pricing policies on health and health economic outcomes in England: an epidemiological model. Lancet. 2010 Apr 17;375(9723):1355-64. doi: 10.1016/S0140-6736(10)60058-X. Epub 2010 Mar 24. PMID: 20338629.
25. Henke RM, Goetzel RZ, McHugh J, Isaac F. Recent experience in health promotion at Johnson & Johnson: lower health spending, strong return on investment. Health Affairs 2011; 30(3): 490–9.
26. Ingels JB, Corso PS,Kogan SM, Brody GH. Cost-effectiveness ofthe strongAfrican American families-teen program: 1-year follow-up. Drug and Alcohol Dependence 2013;133:556–61.
27. Kuklinski MR, Fagan AA, Hawkins JD, Briney JS, Catalano RF. Benefit–cost analysis of a randomized evaluation of communities that care: monetizing intervention effects on the initiation of delinquency and substance use through grade 12. J Exp Criminol 2015; 11(2): 165–92.
28. Long CG, Williams M, Hollin CR. Treating alcohol problems: a study of programme effectiveness and cost effectiveness according to length and delivery of treatment. Addiction. 1998 Apr;93(4):561-71.
29. Miller TR, Hendrie D. Substance Abuse Prevention Dollars and Cents: A CostBenefit Analysis. Center for Substance Abuse Prevention, Substance Abuse and Mental Health Services Administration; 2008
30. Nalpas B, Combescure C, Pierre B, Ledent T, Gillet C, Playoust D, Danel T, Bozonnat MC, Martin S, Balmès JL, Daurès JP. Financial costs of alcoholism treatment programs: a longitudinal and comparative evaluation among four specialized centers. Alcohol Clin Exp Res. 2003 Jan;27(1):51-6.
31. Schramm CJ. Measuring the return on program costs: evaluation of a multiemployer alcoholism treatment program. Am J Public Health 1977; 67(1): 50–1
32. Spoth RL, Guyll M, Day SX. Universal family-focused interventions in alcoholuse disorder prevention: cost-effectiveness and cost-benefit analyses of two interventions. J Stud Alcohol 2002; 63(2): 219–28
33. Tanaree A, Assanangkornchai S, Isaranuwatchai W, Thavorn K, Coyte PC. Integrated treatment program for alcohol related problems in community hospitals, Songkhla province of Thailand: a social return on investment analysis. PLoS One 2019; 14(1): e0209210.
34. Thanh NX, Jonsson E, Moffatt J, Dennett L, Chuck AW, Birchard S. An economic evaluation of the parent-child assistance program for preventing fetal alcohol spectrum disorder in Alberta, Canada. Adm Policy Ment Health 2015; 42(1): 10–8.
35. Angus C, Scafato E, Ghirini S, Torbica A, Ferre F, Struzzo P, et al. Cost-effectiveness of a programme of screening and brief interventions for alcohol in primary care in Italy. BMC Fam Pract 2014; 15: 26.
36. Angus C, Thomas C, Anderson P, Meier PS, Brennan A. Estimating the costeffectiveness of brief interventions for heavy drinking in primary health care across Europe. Eur J Public Health 2017; 27(2): 345–51.
37. Tariq L, van den Berg M, Hoogenveen RT, van Baal PH. Cost-effectiveness of an opportunistic screening programme and brief intervention for excessive alcohol use in primary care. PLoS One 2009;4:e5696.
38. Gentilello LM, Ebel BE, Wickizer TM, Salkever DS, Rivara FP. Alcohol interventions for trauma patients treated in emergency departments and hospitals: a cost benefit analysis. Ann Surg. 2005 Apr;241(4):541-50.
39. Havard A, Shakeshaft AP, Conigrave KM, Doran CM. Randomized controlled trial of mailed personalized feedback for problem drinkers in the emergency department: the short-term impact. Alcoholism: Clinical and Experimental Research 2012;36:523–31
40. Kunz FM Jr, French MT, Bazargan-Hejazi S. Cost-effectiveness analysis of a brief intervention delivered to problem drinkers presenting at an inner-city hospital emergency department. J Stud Alcohol. 2004 May;65(3):363-70.
41. Mundt MP. Analyzing the costs and benefits of brief intervention. Alcohol Res Health 2006; 29(1): 34–6.
42. Navarro HJ, Shakeshaft A, Doran CM, Petrie DJ. The potential cost-effectiveness of general practitioner delivered brief intervention for alcohol misuse: evidence from rural Australia. Addict Behav 2011; 36(12): 1191–8
43. Paltzer J, Moberg DP, Burns M, Brown RL. Health Care Utilization After Paraprofessional-administered Substance Use Screening, Brief Intervention, and Referral to Treatment: A Multi-level Cost-offset Analysis. Med Care. 2019 Sep;57(9):673-679.
44. Pringle JL, Kelley DK, Kearney SM, Aldridge A, Dowd W, Johnjulio W, et al. Screening, brief intervention, and referral to treatment in the emergency department. Med Care 2018; 56(2): 146–52.
45. Purshouse RC, Brennan A, Rafia R, Latimer NR, Archer RJ, Angus CR, et al. Modelling the cost-effectiveness of alcohol screening and brief interventions in primary care in England. Alcohol Alcohol 2013; 48(2): 180–8.
46. Quanbeck A, Lang K, Enami K, Brown RL. A cost-benefit analysis of Wisconsin’s screening, brief intervention, and referral to treatment program: adding the employer’s perspective. WMJ 2010; 109(1): 9–14.
47. Shanahan M, Shakeshaft A, Mattick RP. Modelling the costs and outcomes of changing rates of screening for alcohol misuse by GPs in the Australian context. Applied Health Economics and Health Policy 2006;5:155–66.
48. Zur RM, Zaric GS. A microsimulation cost – utility analysis of alcohol screening and brief intervention to reduce heavy alcohol consumption in Canada. Addiction 2015;111:817–31.
49. Babor TF, Higgins-Biddle JC, Dauser D, Burleson JA, Zarkin GA, Bray J. Brief interventions for at-risk drinking: patient outcomes and cost-effectiveness in managed care organizations. Alcohol Alcohol. 2006 Nov-Dec;41(6):624-31.
50. Barbosa C, Cowell A, Bray J, Aldridge A. The cost-effectiveness of alcohol screening, brief intervention, and referral to treatment (SBIRT) in emergency and outpatient medical settings. Journal of Substance Abuse Treatment 2015;53:1–8.
51. Smit F, Lokkerbol J, Riper H, Majo MC, Boon B, Blankers M. Modeling the cost-effectiveness of health care systems for alcohol use disorders: how implementation of eHealth interventions improves cost-effectiveness. Journal of Medical Internet Research 2011;13:e56.
52. Lock CA, Kaner E, Heather N, Doughty J, Crawshaw A, McNamee P, Purdy S, Pearson P. Effectiveness of nurse-led brief alcohol intervention: a cluster randomized controlled trial. J Adv Nurs. 2006 May;54(4):426-39.
53. Kapoor A, Kraemer KL, Smith KJ, Roberts MS, Saitz R. Cost-effectiveness of screening for unhealthy alcohol use with % carbohydrate deficient transferrin: results from a literature-based decision analytic computer model. Alcohol Clin Exp Res 2009; 33(8): 1440–9.
54. Miller TR, Zaloshnja E, Spicer RS. Effectiveness and benefit-cost of peer-based workplace substance abuse prevention coupled with random testing. Accid Analy Prev 2007; 39(3): 565–73.
55. Cowell AJ, Brown JM, Mills MJ, Bender RH, Wedehase BJ. Cost-effectiveness analysis of motivational interviewing with feedback to reduce drinking among a sample of college students. Journal of Studies on Alcohol and Drugs 2012;73:226–37.
56. Deluca P, Coulton S, Alam MF, et al. Brief interventions to prevent excessive alcohol use in adolescents at low-risk presenting to emergency departments: three-arm, randomised trial of effectiveness and cost-effectiveness. Int J Drug Policy 2021; 93: 103113
57. Downs SM, Klein JD. Clinical preventive services efficacy and adolescents' risky behaviors. Arch Pediatr Adolesc Med. 1995 Apr;149(4):374-9. doi: 10.1001/archpedi.1995.02170160028004. PMID: 7704164.
58. Watson J, Crosby H, Dale V, Tober G, Wu Q, Lang J, et al. AESOPS: a randomised controlled trial of the clinical effectiveness and cost-effectiveness of opportunistic screening and stepped care interventions for older hazardous alcohol users in primary care. Health Technology Assessment 2013;17
59. Byrnes JM, Cobiac LJ, Doran CM, Vos T, Shakeshaft AP. Cost-effectiveness of volumetric alcohol taxation in Australia. Med J Aust 2010; 192(8): 439–43
60. Van den Berg M, van Baal PHM, Tariq L, Schuit AJ, de Wit GA, Hoogenveen RT. The cost-effectiveness of increasing alcohol taxes: a modelling study. BMC Med 2008; 6: 36
61. Cobiac LJ, Mizdrak A, Wilson N. Cost-effectiveness of raising alcohol excise taxes to reduce the injury burden of road traffic crashes. Injury Prevention 2018;0:1–7.
62. Doran CM, Byrnes JM, Cobiac LJ, Vos T, Vandenberg B. Estimated impacts of alternative Australian alcohol taxation structures on consumption, public health and government revenues. Med J Aust 2013; 199(9): 619–22.
63. Holm AL, Veerman L, Cobiac L, Ekholm O, Diderichsen F. Cost-effectiveness of changes in alcohol taxation in Denmark: a modelling study. Cost Eff Resour Alloc 2014; 12(1): 1.
64. Holm AL, Veerman L, Cobiac L, Ekholm O, Diderichsen F. Cost-effectiveness of preventive interventions to reduce alcohol consumption in Denmark. PLoS One 2014;9:e88041.
65. Patra J, Rehm J, Popova S. Avoidable alcohol-attributable criminality and its costs due to selected interventions in Canada. Int J Drug Policy 2011; 22(2): 109–19.
66. Blankers M, Nabitz U, Smit F, Koeter MWJ, Schippers GM. Economic evaluation of internet-based interventions for harmful alcohol use alongside a pragmatic randomized controlled trial. J Med Internet Res 2012; 14(5): e134
67. Brodtkorb TH, Bell M, Irving AH, Laramée P. The Cost Effectiveness of Nalmefene for Reduction of Alcohol Consumption in Alcohol-Dependent Patients with High or Very High Drinking-Risk Levels from a UK Societal Perspective. CNS Drugs. 2016 Feb;30(2):163-77.
68. Coulton S, Bland M, Crosby H, Dale V, Drummond C, Godfrey C, et al. Effectiveness and cost-effectiveness of opportunistic screening and steppedcare interventions for older alcohol users in primary care. Alcohol Alcohol 2017; 52(6): 655–64
69. Cowell AJ, Zarkin GA, Wedehase BJ, Lerch J, Walters ST, Taxman FS. Cost and cost-effectiveness of computerized vs. in-person motivational interventions in the criminal justice system. J Subst Abuse Treat 2018; 87: 42–9.
70. Crawford MJ, Sanatinia R, Barrett B, Byford S, Dean M, Green J, et al. The clinical and cost-effectiveness of brief advice for excessive alcohol consumption among people attending sexual health clinics: a randomised controlled trial. Sexually Transmitted Infections 2015;91:37–43.
71. Ditsuwan V, Lennert Veerman J, Bertram M, Vos T. Cost-effectiveness of interventions for reducing road traffic injuries related to driving under the influence of alcohol. Value Health 2013; 16(1): 23–30
72. Drummond C, Coulton S, James D, Godfrey C, Parrott S, Baxter J, et al. Effectiveness and cost-effectiveness of a stepped care intervention for alcohol use disorders in primary care: pilot study. British Journal of Psychiatry 2009;195:448–56
73. Dunlap LJ, Zarkin GA, Bray JW, Mills M, Kivlahan DR, McKay JR, Latham P, Tonigan JS. Revisiting the cost-effectiveness of the COMBINE study for alcohol dependent patients: the patient perspective. Med Care. 2010 Apr;48(4):306-13.
74. Galárraga O, Gao B, Gakinya BN, Klein DA, Wamai RG, Sidle JE, et al. Task-shifting alcohol interventions for HIV+ persons in Kenya: a cost-benefit analysis. BMC Health Serv Res 2017; 17: 239.
75. Kruger J, Brennan A, Strong M, Thomas C, Norman P, Epton T. The costeffectiveness of a theory-based online health behaviour intervention for new university students: an economic evaluation. BMC Public Health 2014; 14: 1011.
76. Lai T, Habicht J, Reinap M, Chisholm D, Baltussen R. Costs, health effects and cost-effectiveness of alcohol and tobacco control strategies in Estonia. Health Policy (New York) 2007;84:75–88.
77. Laramée P, Bell M, Irving A, Brodtkorb TH. The Cost-Effectiveness of the Integration of Nalmefene within the UK Healthcare System Treatment Pathway for Alcohol Dependence. Alcohol Alcohol. 2016 May;51(3):283-90.
78. Laramée P, Brodtkorb TH, Rahhali N, Knight C, Barbosa C, François C, Toumi M, Daeppen JB, Rehm J. The cost-effectiveness and public health benefit of nalmefene added to psychosocial support for the reduction of alcohol consumption in alcohol-dependent patients with high/very high drinking risk levels: a Markov model. BMJ Open. 2014 Sep 16;4(9):e005376.
79. Millier A, Laramée P, Rahhali N, Aballéa S, Daeppen JB, Rehm J, Toumi M. Cost-Effectiveness of Nalmefene Added to Psychosocial Support for the Reduction of Alcohol Consumption in Alcohol-Dependent Patients With High/Very High Drinking Risk Levels: A Microsimulation Model. J Stud Alcohol Drugs. 2017 Nov;78(6):867-876.
80. Mortimer D, Segal L. Economic evaluation of interventions for problem drinking and alcohol dependence: cost per QALY estimates. Alcohol Alcohol. 2005 Nov-Dec;40(6):549-55.
81. Neighbors CJ, Barnett NP, Rohsenow DJ, Colby SM, Monti PM. Cost-effectiveness of a motivational intervention for alcohol-involved youth in a hospital emergency department. J Stud Alcohol Drugs 2010; 71(3): 384–94.
82. O'Farrell TJ, Choquette KA, Cutter HS, Floyd FJ, Bayog R, Brown ED, Lowe J, Chan A, Deneault P. Cost-benefit and cost-effectiveness analyses of behavioral marital therapy as an addition to outpatient alcoholism treatment. J Subst Abuse. 1996;8(2):145-66.
83. Palmer AJ, Neeser K, Weiss C, Brandt A, Comte S, Fox M. The long-term cost-effectiveness of improving alcohol abstinence with adjuvant acamprosate. Alcohol Alcohol. 2000 Sep-Oct;35(5):478-92.
84. Parrott S, Godfrey C, Heather N, Clark J, Ryan T. Cost and outcome analysis of two alcohol detoxification services. Alcohol Alcohol. 2006 Jan-Feb;41(1):84-91.
85. Pettinati HM, Meyers K, Evans BD, Ruetsch CR, Kaplan FN, Jensen JM, Hadley TR. Inpatient alcohol treatment in a private healthcare setting: which patients benefit and at what cost? Am J Addict. 1999 Summer;8(3):220-33.
86. Rychlik R, Siedentop H, Pfeil T, Daniel D. Cost-effectiveness of adjuvant treatment with acamprosate in maintaining abstinence in alcohol dependent patients. Eur Addict Res. 2003 Apr;9(2):59-64.
87. Schädlich PK, Brecht JG. The cost effectiveness of acamprosate in the treatment of alcoholism in Germany. Economic evaluation of the Prevention of Relapse with Acamprosate in the Management of Alcoholism (PRAMA) Study. Pharmacoeconomics. 1998 Jun;13(6):719-30.
88. Schulz DN, Smit ES, Stanczyk NE, Kremers SPJ, de Vries H, Evers SM. Economic evaluation of a web-based tailored lifestyle intervention for adults: findings regarding cost-effectiveness and cost-utility from a randomized a controlled trial. J Med Internet Res 2014; 16(3): 338–401.
89. Sluiter RL, Kievit W, van der Wilt GJ, Schene AH, Teichert M, Coenen MJH, Schellekens A. Cost-Effectiveness Analysis of Genotype-Guided Treatment Allocation in Patients with Alcohol Use Disorders Using Naltrexone or Acamprosate, Using a Modeling Approach. Eur Addict Res. 2018;24(5):245-254.
90. UKATT Research Team. Cost effectiveness of treatment for alcohol problems: findings of the randomised UK alcohol treatment trial (UKATT). BMJ. 2005 Sep 10;331(7516):544.
91. Walters D, Connor JP, Feeney GF, Young RM. The cost effectiveness of naltrexone added to cognitive-behavioral therapy in the treatment of alcohol dependence. J Addict Dis. 2009;28(2):137-44.
92. Watson JM, Fairhurst C, Li J, Tober G, Crosby H, Lloyd C, et al. ADAPTA: a pilot randomised controlled trial of an alcohol-focused intervention versus a healthy living intervention for problem drinkers identified in a general hospital setting. Drug Alcohol Depend 2015; 154: 117–24.
93. Zarkin GA, Bray JW, Aldridge A, Mitra D, Mills MJ, Couper DJ, Cisler RA; COMBINE Cost-Effectiveness Research Group. Cost and cost-effectiveness of the COMBINE study in alcohol-dependent patients. Arch Gen Psychiatry. 2008 Oct;65(10):1214-21.
94. Alwyn T, John B, Hodgson RJ, Phillips CJ. The addition of a psychological intervention to a home detoxification programme. Alcohol Alcohol. 2004 Nov-Dec;39(6):536-41.
95. Annemans L, Vanoverbeke N, Tecco J, D'Hooghe D. Economic evaluation of Campral (Acamprosate) compared to placebo in maintaining abstinence in alcohol-dependent patients. Eur Addict Res. 2000 Jun;6(2):71-8.
96. Bischof G, Grothues JM, Reinhardt S, Meyer C, John U, Rumpf HJ. Evaluation of a telephone-based stepped care intervention for alcohol-related disorders: a randomized controlled trial. Drug Alcohol Depend. 2008 Mar 1;93(3):244-51.
97. Fals-Stewart W, Klostermann K, Yates BT, O'Farrell TJ, Birchler GR. Brief relationship therapy for alcoholism: a randomized clinical trial examining clinical efficacy and cost-effectiveness. Psychol Addict Behav. 2005 Dec;19(4):363-71.
98. Shepard DS, Lwin AK, Barnett NP, Mastroleo N, Colby SM, Gwaltney C, et al. Cost-effectiveness of motivational intervention with significant others for patients with alcohol misuse. Addiction 2016; 111(5): 832–9.
99. Avanceña ALV, Miller N, Uttal SE, Hutton DW, Mellinger JL. Cost-effectiveness of alcohol use treatments in patients with alcohol-related cirrhosis. J Hepatol. 2021 Jun;74(6):1286-1294.
100. Ministry of Health and Family Welfare. Government of India. Mental, neurological, and substance use disorders care at Health and Wellness Centres. Operational Guidelines. Available from: <https://aam.mohfw.gov.in/download/document/Final_MNS_Operational_Guidelines_-_Web_Optimized_PDF_Version_-_19_11_20.pdf>, last accessed on 14.05.2025.
101. Ambekar A, Agrawal A, Rao R, Mishra AK, Khandelwal SK, Chadda RK on behalf of the group of investigators for the National Survey on Extent and Pattern of Substance Use in India (2019). Magnitude of Substance Use in India. New Delhi: Ministry of Social Justice and Empowerment, Government of India.
102. Nadkarni A, Garber A, Costa S, Wood S, Kumar S, MacKinnon N, Ibrahim M, Velleman R, Bhatia U, Fernandes G, Weobong B, Rane A. Auditing the AUDIT: A systematic review of cut-off scores for the Alcohol Use Disorders Identification Test (AUDIT) in low- and middle-income countries. Drug Alcohol Depend. 2019 Sep 1;202:123-133. doi: 10.1016/j.drugalcdep.2019.04.031. Epub 2019 Jul 16. PMID: 31349205.
103. Gururaj G, Varghese M, Benegal V, Rao GN, Pathak K, Singh LK, Mehta RY, Ram D, Shibukumar TM, Kokane A, Lenin Singh RK, Chavan BS, Sharma P, Ramasubramanian C, Dalal PK, Saha PK , Deuri SP, Giri AK, Kavishvar AB, Sinha VK, Thavody J, Chatterji R, Akoijam BS, Das S, Kashyap A, Ragavan VS, Singh SK, Misra R and NMHS collaborators group. National Mental Health Survey of India, 2015-16: Mental Health Systems. Bengaluru, National Institute of Mental Health and Neuro Sciences, NIMHANS Publication No. 130, 2016.
104. Ministry of Health and Family Welfare. Government of India. Management of Alcohol Dependence. Standard Treatment Guidelines. 2016. Available from: <https://nhm.gov.in/images/pdf/guidelines/nrhm-guidelines/stg/Alcohol_Dependence.pdf>, last accessed on 06.01.2024.
105. Kuchya S, Kumar A. A longitudinal study to assess adherence towards de-addiction treatment in alcohol addicted patients. Indian Journal of Applied Research. 2019;9(2):49-50

106. Shah N, Karia S, Gowda M, Gupta G, Dey S, Mulakaluri PP, Sidana A, Pangaonkar S. Assessment of treatment patterns in patients with alcohol withdrawal syndrome during hospitalization and post-discharge: A retrospective cohort study. Journal of Clinical and Diagnostic Research. 2023;17(9):VC01-VC06

107. Gopaldas JA, Padyana M, Rai PP. Practice patterns in the diagnosis and management of alcohol withdrawal syndrome in Indian Intensive Care Units. 2023; 27(11):816-820.

108. Ballesteros J, Duffy JC, Querejeta I, Arino J, Gonzalez-Pinto A. Efficacy of Brief Interventions for Hazardous Drinkers in Primary Care: Systematic Review and Meta-Analyses. Alcoholism: Clinical & Experimental Research. 2004; 28(4): 608–618.

109. de Sousa A, de Sousa A. An open randomized study comparing disulfiram and acamprosate in the treatment of alcohol dependence. Alcohol Alcohol. 2005 Nov-Dec;40(6):545-8. doi: 10.1093/alcalc/agh187. Epub 2005 Jul 25. PMID: 16043433.
110. De Sousa A, De Sousa A. A one-year pragmatic trial of naltrexone vs disulfiram in the treatment of alcohol dependence. Alcohol Alcohol. 2004 Nov-Dec;39(6):528-31. doi: 10.1093/alcalc/agh104. PMID: 15525790.
111. De Sousa AA, De Sousa J, Kapoor H. An open randomized trial comparing disulfiram and topiramate in the treatment of alcohol dependence. J Subst Abuse Treat. 2008 Jun;34(4):460-3.
112. Addolorato G, Caputo F, Capristo E, Domenicali M, Bernardi M, Janiri L, Agabio R, Colombo G, Gessa GL, Gasbarrini G. Baclofen efficacy in reducing alcohol craving and intake: a preliminary double-blind randomized controlled study. Alcohol Alcohol. 2002 Sep-Oct;37(5):504-8.
113. Prinja et al. Costing of mental health services at tertiary care institution in India. 2025. Unpublished study. [Personal Communication]

114. National Health System Cost Database from India. Available from: <https://www.healtheconomics.pgisph.in/costing_web/index.php?action=gen_output>, last accessed on 06.01.2024

115. Central Government Health Scheme Rates. 2024. Available from: <https://cghs.gov.in/CghsGovIn/faces/ViewPage.xhtml?id=NTQzNg==>, last accessed on 06.01.2024

116. Tata 1 mg. Complete hemogram. Available from: <https://www.1mg.com/labs/test/complete-hemogram-cbc-esr-1743?wpsrc=Google+Organic+Search>, last accessed on 30.12.2024.
117. Redcliffe Labs. Liver Function test. Available from: https://redcliffelabs.com/, last accessed on 30.12.2024.
118. Orange Health Labs. Kidney Function Test (KFT). Available from: <https://www.orangehealth.in/kidney-function-test?utm_term=kidney-function-test&utm_source=seo_search&utm_campaign=seo&utm_medium=google>, last accessed on 30.12.2024.
119. Healthians scans. Available from: https://www.healthians.com/scans, last accessed on 30.12.2024.
120. Indiamart. Available from: <https://www.indiamart.com/>, last accessed on 30.12.2024.
121. Ministry of Statistics & Programme Implementation, Government of India. India - Household Social Consumption: Health, NSS 75th Round Schedule-25.0: July 2017-June 2018. Available from: <http://microdata.gov.in/nada43/index.php/catalog/152>, last accessed on 22.10.2024

122. Ministry of Home Affairs. Government of India. SRS based abridged life tables 2016-20. Available from: <https://censusindia.gov.in/nada/index.php/catalog/44377>, , last accessed on 06.01.2024

123. Ministry of Road Transport and Highways. Government of India. Road accidents in India. 2022. Available from: <https://morth.nic.in/sites/default/files/RA_2022_30_Oct.pdf>
124. Singh P, Lakshmi PVM, Prinja S, Khanduja P. Under-reporting of road traffic accidents in traffic police records- a cross sectional study from North India. International Journal of Community Medicine and Public Health. 2018;5(2):579
125. Gururaj G. Road traffic deaths, injuries and disabilities in India: Current scenario. The National Medical Journal of India. 2008;21(1):14-20
126. Salottolo K, Panchal R, Madayag RM, Dhakal L, Rosenberg W, Banton KL, Hamilton D, Bar-Or D. Incorporating age improves the Glasgow Coma Scale score for predicting mortality from traumatic brain injury. BMJ Trauma Surgery and Acute Care Open. 2021;6:e000641
127. Prinja S, Jagnoor J, Sharma D, Aggarwal S, Katoch S, Lakshmi PVM, Ivers R. Out-of-pocket expenditure and catastrophic health expenditure for hospitalization due to injuries in public sector hospitals in North India. PLoS One. 2019 Nov 7;14(11):e0224721. doi: 10.1371/journal.pone.0224721. PMID: 31697781; PMCID: PMC6837486.
128. Jagnoor J, Prinja S, Nguyen H, Gabbe BJ, Peden M, Ivers RQ. Mortality and health-related quality of life following injuries and associated factors: a cohort study in Chandigarh, North India. Inj Prev. 2020 Aug;26(4):315-323. doi: 10.1136/injuryprev-2019-043143. Epub 2019 Jul 4. PMID: 31273029.
129. Nadkarni A, Weiss HA, Naik A, Bhat B, Patel V. The six-year outcome of alcohol use disorders in men: A population based study from India. Drug Alcohol Depend. 2016 May 1;162:107-15. doi: 10.1016/j.drugalcdep.2016.02.039. Epub 2016 Mar 4. PMID: 26994665; PMCID: PMC4841788.
130. Esser MB, Wadhwaniya S, Gupta S, Tetali S, Gururaj G, Stevens KA, Hyder AA. Characteristics associated with alcohol consumption among emergency department patients presenting with road traffic injuries in Hyderabad, India. Injury. 2016 Jan;47(1):160-5. doi: 10.1016/j.injury.2015.07.022. Epub 2015 Jul 30. PMID: 26260623.
131. Ram B, Thakur R. Measuring the burden of accidental injuries in India: a cross-sectional analysis of the National Sample Survey (2017–18). *Humanit Soc Sci Commun. 2022;* 9:363. <https://doi.org/10.1057/s41599-022-01369-0>
132. Sangwan A, Prinja S, Aggarwal S, Jagnoor J, Bahuguna P, Ivers R. Cost of Trauma Care in Secondary- and Tertiary-Care Public Sector Hospitals in North India. Appl Health Econ Health Policy. 2017 Oct;15(5):681-692. doi: 10.1007/s40258-017-0329-7. PMID: 28409489.
133. Kumar RP, Fathima FN, Agrawal T, Misquith D, Gururaj G. Economic burden of road traffic injuries among hospitalized subjects in a tertiary care center in Bengaluru, India: A cost of illness study. International Journal of Noncommunicable Diseases 8(1):p 21-30, Jan–Mar 2023. | DOI: 10.4103/jncd.jncd_61_22

134. Delacôte C, Bauvin P, Louvet A, Dautrecque F, Ntandja Wandji LC, Lassailly G, Voican C, Perlemuter G, Naveau S, Mathurin P, Deuffic-Burban S. A Model to Identify Heavy Drinkers at High Risk for Liver Disease Progression. Clin Gastroenterol Hepatol. 2020 Sep;18(10):2315-2323.e6. doi: 10.1016/j.cgh.2019.12.041.

135. Julien J, Ayer T, Bethea ED, Tapper EB, Chhatwal J. Projected prevalence and mortality associated with alcohol-related liver disease in the USA, 2019-40: a modelling study. Lancet Public Health. 2020 Jun;5(6):e316-e323. doi: 10.1016/S2468-2667(20)30062-1. PMID: 32504584.
136. National Health Authority. Package Master- HBP 2022. Available from: <https://ayushmanup.in/assets/doc/HBP-2022.pdf>, last accessed on 04.01.2024
137. Prinja S, Bahuguna P, Duseja A, Kaur M, Chawla YK. Cost of Intensive Care Treatment for Liver Disorders at Tertiary Care Level in India. Pharmacoecon Open. 2018 Jun;2(2):179-190. doi: 10.1007/s41669-017-0041-4. PMID: 29623618; PMCID: PMC5972113.
138. Dixit J, Gupta N, Kataki A, Roy P, Mehra N, Kumar L, Singh A, Malhotra P, Gupta D, Goyal A, Rajsekar K, Krishnamurthy MN, Gupta S, Prinja S. Health-related quality of life and its determinants among cancer patients: evidence from 12,148 patients of Indian database. Health Qual Life Outcomes. 2024 Mar 13;22(1):26. doi: 10.1186/s12955-024-02227-0. PMID: 38481231; PMCID: PMC10938809.
139. Che YH, You J, Chongsuvivatwong V, Li L, Sriplung H, Yan YZ, Ma SJ, Zhang X, Shen T, Chen HM, Rao SF, Zhang RY. Dynamics and liver disease specific aspects of quality of life among patients with chronic liver disease in Yunnan, China. Asian Pac J Cancer Prev. 2014;15(12):4765-71. doi: 10.7314/apjcp.2014.15.12.4765. PMID: 24998539.
140. Sher DJ, Tishler RB, Annino D, Punglia RS. Cost-effectiveness of CT and PET-CT for determining the need for adjuvant neck dissection in locally advanced head and neck cancer. Ann Oncol. 2010 May;21(5):1072-7. doi: 10.1093/annonc/mdp405. Epub 2009 Oct 15. PMID: 19833820; PMCID: PMC2895302.
141. Simons PA, Ramaekers B, Hoebers F, Kross KW, Marneffe W, Pijls-Johannesma M, Vandijck D. Cost-Effectiveness of Reduced Waiting Time for Head and Neck Cancer Patients due to a Lean Process Redesign. Value Health. 2015 Jul;18(5):587-96. doi: 10.1016/j.jval.2015.04.003. Epub 2015 Jun 10. PMID: 26297086.
142. Bagal S, Budukh A, Thakur JS, Dora T, Qayyumi B, Khanna D, Fernandes D, Chakravarti P, Singh R, Patil S, Dikshit R, Chaturvedi P. Head and neck cancer burden in India: an analysis from published data of 37 population-based cancer registries. Ecancermedicalscience. 2023 Sep 21;17:1603. doi: 10.3332/ecancer.2023.1603.
143. Znaor A, Brennan P, Gajalakshmi V, Mathew A, Shanta V, Varghese C, Boffetta P. Independent and combined effects of tobacco smoking, chewing and alcohol drinking on the risk of oral, pharyngeal and esophageal cancers in Indian men. International Journal of Cancer. 2003; 105(5:)681686. doi:10.1002/ijc.11114
144. Subramanian S, Sankaranarayanan R, Bapat B, Somanathan T, Thomas G, Mathew B, Vinoda J, Ramadas K. Cost-effectiveness of oral cancer screening: results from a cluster randomized controlled trial in India. Bull World Health Organ. 2009 Mar;87(3):200-6. doi: 10.2471/blt.08.053231.
145. Lokhande M, Kannusamy S, Oak A, Cheulkar S, Chavan S, Mishra V, Gode P, Thakadiyil AS, Mendhe S, Kadam S, Balasubramaniam G, Chaturvedi P, Dikshit R. A hospital-based study of survival in oral cancer
146. Lu HJ, Chiu YW, Lan WS, et al. Prediction Model of Distant Metastasis in Oral Cavity Squamous Cell Carcinoma With or Without Regional Lymphatic Metastasis. Front Oncol. 2021;11:713815.
147. Wong LY, Wei WI, Lam LK, Yuen AP. Salvage of recurrent head and neck squamous cell carcinoma after primary curative surgery. Head Neck. 2003;25(11):953-959.
148. Husereau D, Drummond M, Augustovski F, Briggs AH, Carswell C, Caulley L, Chaiyakunapruk N, de Bekker-Grob E, Greenberg D, Loder E, Mauskopf J, Mullins CD, Petrou S, Pwu RF, Staniszewska S; CHEERS 2022 ISPOR Good Research Practices Task Force. Consolidated Health Economic Evaluation Reporting Standards 2022 (CHEERS 2022) statement: updated reporting guidance for health economic evaluations. BJOG. 2022 Feb;129(3):336-344. doi: 10.1111/1471-0528.17012. PMID: 35014160.
